# Urinary Proteome Characterization of Stroke-Prone Spontaneously Hypertensive Rats

**DOI:** 10.3390/ijms26010021

**Published:** 2024-12-24

**Authors:** Wenshu Meng, Youhe Gao

**Affiliations:** 1 School of Life Sciences and Medicine, Shandong University of Technology, Zibo 255000, China; mengwenshu_sdut@163.com; 2 Department of Biochemistry and Molecular Biology, Gene Engineering Drug and Biotechnology Beijing Key Laboratory, Beijing Normal University, Beijing 100875, China

**Keywords:** hypertension, urine, proteome, SHRSP

## Abstract

Hypertension is a multifactorial and complex disease influenced by genetic and environmental factors, and it has become one of the most serious public health challenges. This study aimed to investigate the changes in hypertension based on urinary proteome. The stroke-prone spontaneously hypertensive rats (SHRSPs) model was used to examined urinary proteome changes during the development of hypertension. Urine proteome profiling was conducted at months 1, 4, 8, 10, 12, and 14 using liquid chromatography coupled with tandem mass spectrometry (LC–MS/MS). Given that the progression of hypertension may vary among individuals, each rat was compared before and after hypertension developed to screen for differential proteins. Differential proteins in each rat can be enriched into some important biological processes and pathways associated with hypertension, such as the regulation of systemic arterial blood pressure by renin-angiotensin, renin-angiotensin signaling, response to glucocorticoid and glucocorticoid receptor signaling, calcium transport I, aldosterone adipocyte signaling pathway, apelin adipocyte signaling pathway, and oxidative stress response. The biological processes and pathways enriched at the same time point in the progression of hypertension differed significantly among different rat individuals. This study demonstrated that the changes in hypertension can be reflected in urine proteins. Urinary proteomics has potential in researching the mechanisms underlying hypertension, discovering new drug targets, and developing personalized strategies for antihypertensive treatment.

## 1. Introduction

Hypertension is a clinical syndrome primarily characterized by elevated systemic arterial blood pressure in the systemic circulation. Over the past thirty years, the global prevalence of hypertension has reached 1.3 billion individuals, making it a major public health challenge [1,2]. Hypertension is directly responsible for over 8.5 million deaths worldwide each year and is a leading risk factor for stroke, ischemic heart disease, and kidney disorders [3,4,5]. Effective treatment and management of hypertension can significantly reduce the incidence and mortality rates associated with blood pressure-related diseases [6]. The main mechanisms underlying hypertension include the sympathetic nervous system, the renal and adrenal function, the endothelium, and insulin resistance [7,8].

Despite the availability of various antihypertensive agents, the prevalence of hypertension and the associated mortality from its complications continue to rise, suggesting that critical pathophysiological mechanisms of hypertension are still not well understood [9]. On the one hand, individual differences in drug responses lead to unstable therapeutic outcomes, complicating the clinical choice of antihypertensive medications. On the other hand, there is a lack of comprehensive research on the pathological mechanisms at different stages of hypertension progression. Therefore, novel methods are urgently needed to better elucidate the pathogenesis of hypertension and to develop personalized treatment strategies for improving blood pressure control.

Proteomics can study endogenous changes and the impact of the external environment on pathophysiological processes as a whole, serving as an effective tool for discovering disease biomarkers, exploring mechanisms of disease, and screening potential drug therapy targets [10]. Without homeostatic regulation, urine can sensitively reflect changes in the body at an early stage [11]. Urine is a promising resource for biomarkers research, and urine proteomics has been applied to the clinical research of multiple diseases, such as gastric cancer [12], lung cancer [13], pediatric medulloblastoma [14], and Parkinson’s disease [15]. Recent studies have shown that urinary proteome has potential for application in the field of cardiovascular disease [16]. For example, by comparing urinary samples from patients with carotid artery stenosis (CAD) to healthy controls, urinary biomarkers have been identified for early screening and risk stratification of CAD [17]. In addition, a urinary proteomic biomarker model has been developed to assist in the diagnosis of acute stroke in those with mild symptoms [18]. As a completely non-invasive sample, a urine sample is very suitable for collection at various stages of disease progression to dynamically monitor a disease. However, the number of proteomic studies for primary hypertension is relatively limited. In this research, we aimed to determine whether the urine proteome can reflect changes associated with hypertension.

The urine proteome is easily affected by external factors, such as age, diet, exercise, sex, medication, and daily rhythms [19]. Animal models can minimize the impact of many uncertain factors, thereby establishing a direct relationship between the disease and corresponding urinary changes. The stroke-prone spontaneously hypertensive rats (SHRSPs) model is a valuable animal model to investigate human hypertension. In this model, the elevation of blood pressure in rats is determined by polygenic inheritance, which is similar to human hypertension [20,21,22,23].

**Figure 1:**
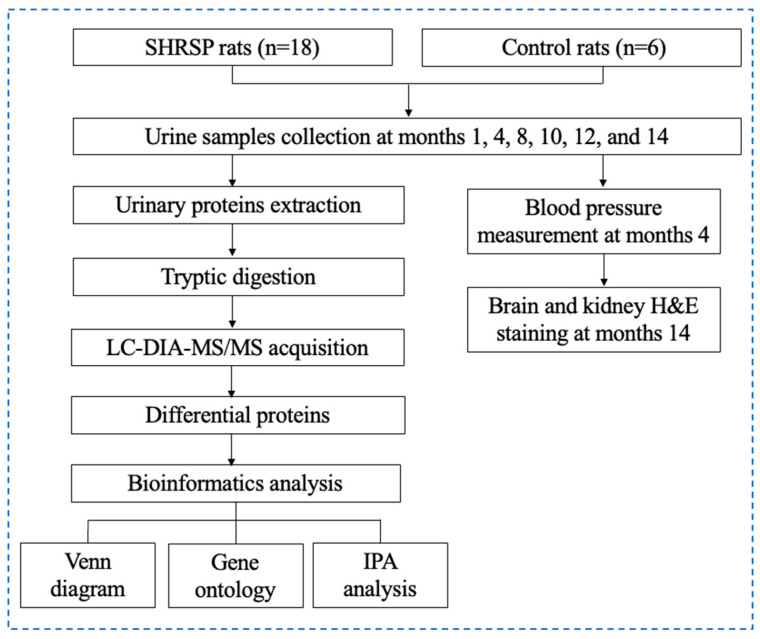
Workflow of the study of urinary proteomics changes in SHRSP rats.

In this study, we employed the SHRSP model to simulate the occurrence and progression of hypertension and intend to dynamically explore the pathological mechanisms of hypertension based on data-independent acquisition (DIA) technology. The workflow of this study is presented in Figure 1.

## 2. Results

### 2.1. Characterization of SHRSP Rats

**Figure 2:**
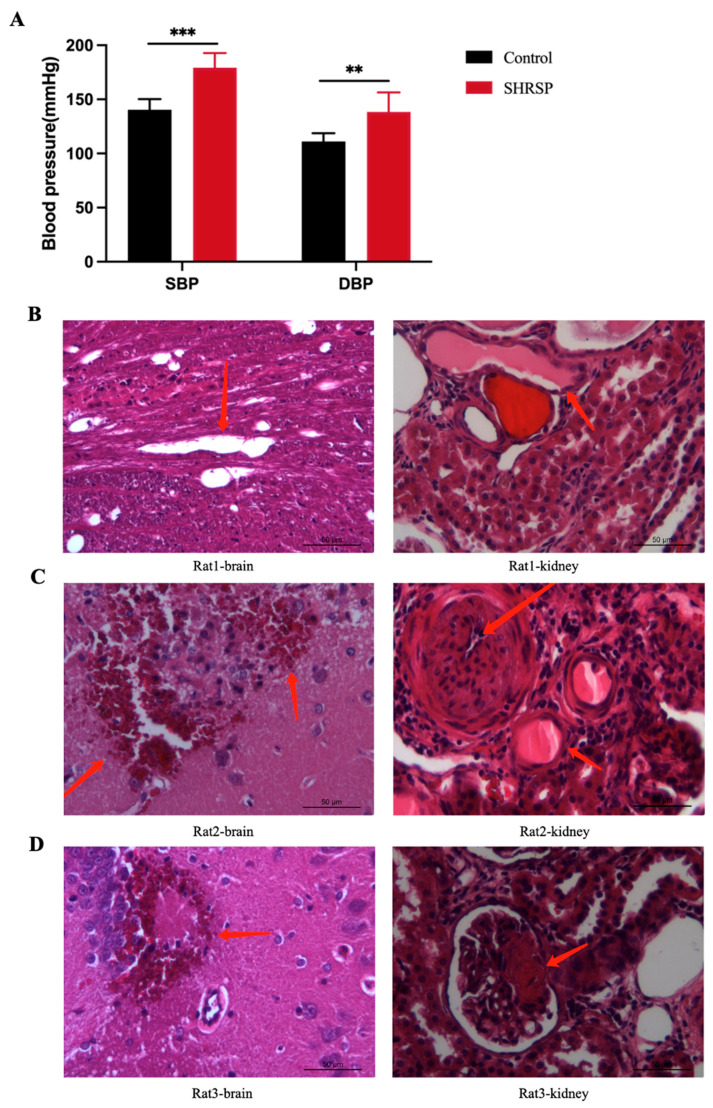
Blood pressure and histopathology in a SHRSP rat model. (**A**) Blood pressure of the SHRSP rat model at 4 months. SHRSP represents the experimental group (*n* = 18). Control represents the control group (*n* = 6). SBP is systolic blood pressure, and DBP is diastolic blood pressure. ** *p* < 0.01 and *** *p* < 0.01. (**B**–**D**) Histopathological characterization of the brain and kidney tissue in the SHRSP model. H&E staining, 20 times magnification. The arrows in (**B**) point to cavities in the brain tissue and protein casts in the renal tubules; the arrows in (**C**) point to periventricular hemorrhage, protein casts, and fibrosis in the renal tubules; the arrows in (**D**) point to periventricular hemorrhage and protein casts in the renal tubules.

The blood pressure measurements were taken to assess if there were any differences between SHRSP control rats by the tail cuff method. The systolic blood pressure (SBP) and the diastolic blood pressure (DBP) showed significant differences between the SHRSP (179 ± 13.1 mmHg systole; 138 ± 17.4 mmHg diastole) and control rats (140 ± 9.0 mmHg systole; 111 ± 6.9 mmHg diastole) at month 4 (Figure 2A). Both SBP and DBP in SHRSP rats were markedly significant compared to the control rats. At month 14, the SHRSPs exhibited prone position without movement, inability to walk, and depression-like behavior. Histopathological examination was performed on the brains and kidneys of three rats. As shown in Figure 2B, Rat 1 showed no observable changes in the brain, with only minimal swelling, degeneration, and fibrosis in some of the glomeruli and tubules. In Rat 2, hemorrhagic foci were visible around the hippocampus and ventricles, with necrosis and atrophy in the glomeruli and tubules, some glomerular hyaline degeneration and fibrosis, thickened arteriole walls, and inflammatory cell infiltration. In Rat 3, multiple scattered hemorrhagic foci were found in the cortical and hippocampal regions of the brain, with necrosis and atrophy in the glomeruli and tubules, some glomerular hyaline degeneration and fibrosis, thickened arteriolar walls, and multiple infiltrating inflammatory cells around narrowed lumens. The SHRSP rats exhibited clinical characteristics of elevated blood pressure, along with varying degrees of cerebral lesions and renal pathology. Overall, the SHRSP rat model mimics the onset and progression of hypertension.

### 2.2. Urinary Proteome Changes in SHRSP Rats

To investigate the urine proteome changes in the progression with hypertension, urine samples from four SHRSP rats and from six time points (months 1, 4, 8, 10, 12, and 14) were analyzed by label-free DIA–MS/MS quantitation. A total of 884 urinary proteins were identified with FDR < 0.01. After the missing values of proteomic data were imputed with the sequential-KNN method, a total of 628 proteins were retained for subsequent differential urinary protein selection. Considering the complexity of hypertension and that disease progression and clinical manifestations of individuals might be different, the self-comparison approach was adopted to screen for differential proteins. The screening criteria was as follows: fold change ≥ 2 or ≤0.5, *p* < 0.05.

**Figure 3:**
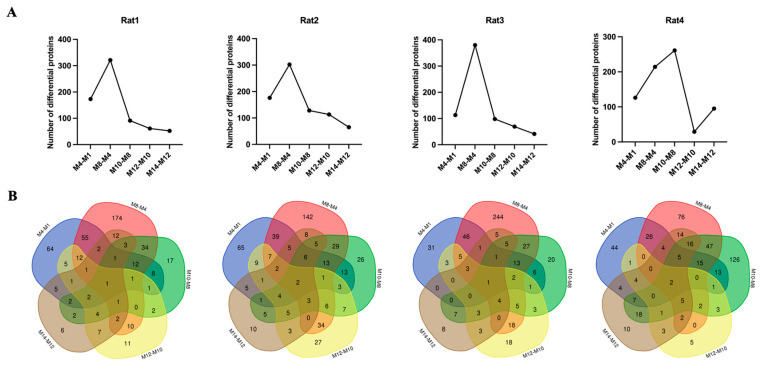
Proteomics changes of urine samples in a SHRSP rat model. (**A**) Changes in the number of differential urinary proteins identified at multiple time points in SHRSP rats. (**B**) Venn diagrams of the differential urinary proteins identified at multiple time points in SHRSP rats.

For Rat1, a total of 173, 321, 91, 61, and 52 differential proteins were significantly changed at months 4, 8, 10, 12, and 14, respectively (Figure 3A, Table S1). The overlap of these differential proteins screened at five time points is presented in Figure 3B, where only one protein was consistently identified across all time points. For Rat2, a total of 176, 302, 128, 113, and 65 differential proteins were significantly changed at months 4, 8, 10, 12, and 14, respectively (Figure 3A, Table S1). The overlap of these differential proteins screened at five time points is presented in Figure 3B, with only two proteins overlapping across all time points. For Rat3, a total of 113, 380, 98, 69, and 41 differential proteins were significantly changed at months 4, 8, 10, 12, and 14, respectively (Figure 3A, Table S1). The overlap of these differential proteins screened at five time points is presented in Figure 3B, with one protein identified across all five time points. For Rat4, a total of 126, 214, 261, 29, and 95 differential proteins were significantly changed at months 4, 8, 10, 12, and 14, respectively (Figure 3A, Table S1). The overlap of these differential proteins screened at five time points is presented in Figure 3B, with two proteins showing overlap across all time points. These results indicated that the majority of differential proteins changed uniquely at multiple different time points, suggesting that different biological changes may occur during the progression of hypertension.

### 2.3. Functional Analysis of Differential Proteins in SHRSP Rats

To explore the changes in pathophysiological functions at different stages in the progression of hypertension, functional annotation was performed on the differential proteins identified at months 4, 8, and 14, including the biological processes and pathways changes.

**Figure 4:**
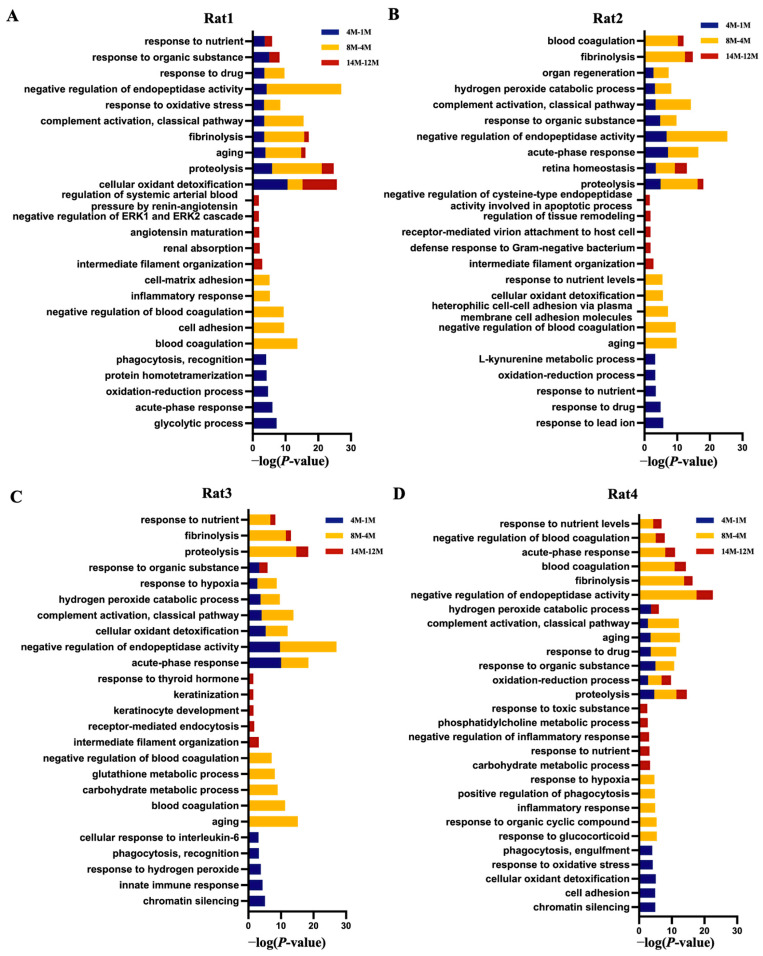
Biological processes enrichment analysis of differential proteins in the progression of hypertension in SHRSP rats. (**A**) Rat 1 (**B**) Rat 2 (**C**) Rat 3 (**D**) Rat 4. The top 5 biological processes of differential proteins at multiple time points in SHRSP rats. All enrichment items were consistent with the *p* < 0.05 standard.

**Figure 5:**
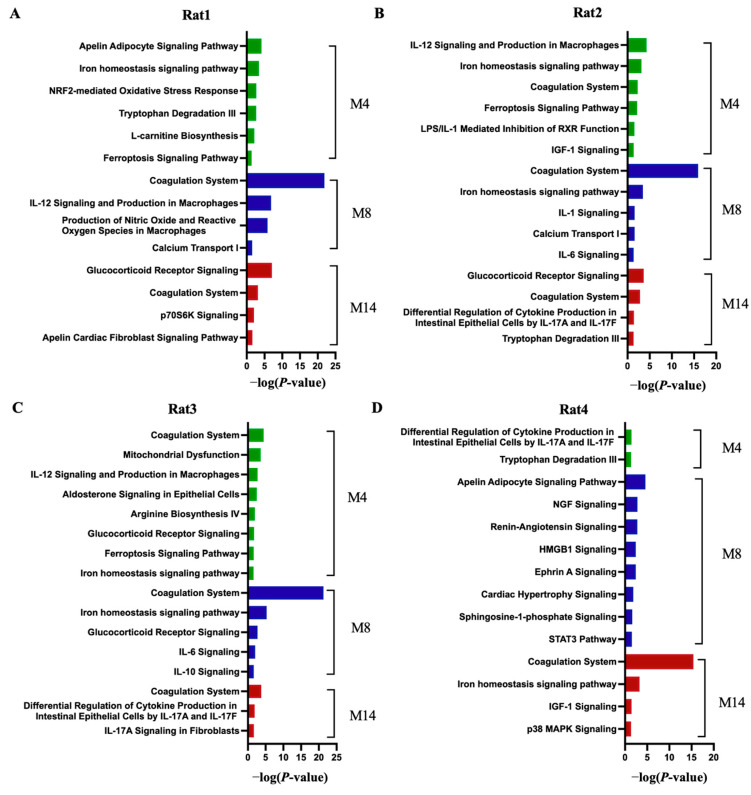
Canonical pathway enrichment analysis of differential proteins in the progression of hypertension in SHRSP rats. (**A**) Rat 1 (**B**) Rat 2 (**C**) Rat 3 (**D**) Rat 4. The representative pathways of differential proteins at multiple time points in SHRSP rats. All enrichment items were consistent with the *p* < 0.05 standard.

In Rat 1, the biological process category (Figure 4A) revealed that cellular oxidant detoxification and proteolysis were mainly enriched at all time points, and the glycolytic process was only enriched at month 4, blood coagulation and inflammatory response were only enriched at month 8, and the regulation of systemic arterial blood pressure by renin-angiotensin was only enriched at month 14. In the pathway category (Figure 5A), apelin adipocyte signaling pathway, iron homeostasis signaling pathway, and NRF2-mediated oxidative stress response were enriched at month 4; coagulation system and calcium transport I were enriched at month 8; and glucocorticoid receptor signaling was enriched at month 14.

In Rat 2, the biological process category (Figure 4B) showed that proteolysis and retina homeostasis were mainly enriched at all time points, the response to lead ion was only enriched at month 4, the negative regulation of blood coagulation was only enriched at month 8, and intermediate filament organization and defense response to Gram-negative bacterium were only enriched at month 14. In the pathway category (Figure 5B), IL-12 signaling and production in macrophages and IGF-1 signaling were enriched at month 4; IL-1 signaling, calcium transport I, and IL-6 signaling were enriched at month 8; and glucocorticoid receptor signaling was enriched at month 14.

In Rat 3, the biological process category (Figure 4C) revealed that acute-phase response and complement activation classical pathway were enriched at months 4 and 8, chromatin silencing and innate immune response were only enriched at month 4, aging and blood coagulation were only enriched at month 8, and intermediate filament organization was only enriched at month 14. In the pathway category (Figure 5C), coagulation system was enriched at all time points, glucocorticoid receptor signaling was enriched at months 4 and 8, IL-10 signaling was enriched at month 8, and IL-17A signaling in fibroblasts was enriched at months14.

In Rat 4, the biological process category (Figure 4D) showed that proteolysis and the oxidation-reduction process were enriched at all time points, chromatin silencing and cell adhesion were only enriched at month 4, the response to glucocorticoid was only enriched at month 8, and the carbohydrate metabolic process was only enriched at month 14. In the pathway category (Figure 5D), tryptophan degradation III was enriched at month 4, renin-angiotensin signaling and the STAT3 pathway were enriched at month 8, and p38 MAPK signaling was enriched at month 14.

Overall, the above results showed that the progression of hypertension can be reflected in urinary proteome changes, and the biological processes and pathways enriched at the same time point differed significantly among different rat individuals.

## 3. Discussion

Hypertension, a multifactorial disease involving environmental and genetic factors, as well as risky behaviors, has emerged as one of the most critical and expensive public health challenges. Despite extensive research, the pathophysiological mechanisms underlying hypertension remain poorly understood. In this study, we systematically investigated dynamic changes in the urinary proteome of SHRSP rats throughout hypertension development.

Several enriched biological functions and pathways identified in SHRSP rats were linked to the pathophysiological mechanisms of hypertension or potential drug targets. For example, (i) the regulation of systemic arterial blood pressure by renin-angiotensin and renin-angiotensin signaling was enriched in the SHRSP rats. Systemic arterial hypertension is characterized by persistently high BP in the systemic arteries [24]. Blockers of the renin–angiotensin–aldosterone system (RAAS) is the cornerstone in the treatment of hypertension [25]. (ii) The response to glucocorticoid and glucocorticoid receptor signaling were enriched in the SHRSP rats. Glucocorticoids participate in regulating blood pressure through various extrarenal tissues. Excess glucocorticoid levels cause promiscuous activation of mineralocorticoid receptors and induce hypertension. Glucocorticoid receptors are widely expressed in many organ systems involved in blood pressure regulation and play an important role in the pathogenesis and maintenance of hypertension [26]. (iii) Calcium Transport I was enriched in SHRSP rats. Calcium channel blockers (CCBs) inhibit the flow of extracellular calcium through ion-specific channels across the cell membrane. Although several types of calcium channels have been identified, currently available CCBs inhibit L-type channels in humans. When the inward calcium flow is inhibited, vascular smooth muscle cells relax, leading to vasodilation and lower BP [27]. (iv) The aldosterone adipocyte signaling pathway was enriched in SHRSP rats. Aldosterone is a steroid hormone that is synthesized and secreted by the glomerular zona in the outer layer of the adrenal cortex, regulates sodium homeostasis, and controls blood volume and blood pressure. Excess secretion of this hormone may lead to hypertension and exacerbate disease morbidity and mortality. An understanding of the signaling pathway that regulates aldosterone biosynthesis may help researchers identify new targets for therapeutic intervention in cardiovascular diseases such as hypertension [28]. (v) The apelin adipocyte signaling pathway was enriched with SHRSP rats. Apelin is a vasoactive peptide, and its receptor APJ is widely expressed in the cardiovascular regulatory regions of blood vessels, heart, and brain in the cardiovascular system. Based on accumulating evidence, the apelin/APJ receptor system plays a regulatory role in cardiovascular physiology and pathophysiology, making it a potential target for cardiovascular drug discovery and development [29]. (vi) The oxidative stress response was enriched in SHRSP rats. Studies have shown that oxidative stress is involved in the pathogenesis of hypertension [30], and oxidative stress promotes endothelial dysfunction, vascular remodeling, and inflammation in the pathophysiological process of hypertension, leading to vascular damage [31]. Clinical studies of patients with essential hypertension have shown that blood pressure is positively correlated with the levels of oxidative stress biomarkers and negatively correlated with the antioxidant levels [32]. Urinary proteomics will increase our understanding of the pathogenesis and progression of hypertension and will someday be used to monitor the progression of hypertension.

The correlation between the chronology of hypertension onset and age presents a formidable challenge in the execution of long-term longitudinal human studies. It is crucial to acknowledge that disease progression varies between individuals. Consequently, proteomic analyses of multifactorial complex diseases can be effectively conducted through comparative assessments of individual. Concurrently, to mitigate the impact on growth and developmental processes, the present study implemented differential protein screening by examining the time points preceding and following the onset of hypertension in a single rat. Our findings revealed several significant functional annotations in spontaneously hypertensive rats (SHRSPs), suggesting that a limited number of important pathways may be involved in hypertension. Furthermore, we observed that, despite sharing the hypertensive condition, distinct individuals may be engaged in divergent pathological mechanisms during the emergence and progression of hypertension. The distinct biological pathways identified in individual rats could potentially offer novel perspectives on the personalized diagnosis and treatment of hypertension. In the future, pathway analysis in hypertensive patients could be facilitated through urinary proteome analysis, enabling the selection of targeted, personalized therapeutics.

## 4. Materials and Methods

### 4.1. Experimental Animals and Model Establishment

Male SHRSPs (*n* = 18, 140 ± 20 g) and male Wistar rats aged 4 weeks (n = 6, 140 ± 20 g) were purchased from Beijing Vital River Laboratory Animal Technology Co., Ltd. (Beijing, China). Animals were fed a standard laboratory diet and housed under controlled indoor temperature (21 ± 2 °C), humidity (65–70%), and 12 h/12 h light–dark cycle conditions. The study was approved by Peking Union Medical College (Approval ID: ACUC-A02-2014-007) and performed according to the guidelines developed by the Institutional Animal Care and Use Committee.

### 4.2. Urine Collection and Sample Preparation

Urine samples were collected from the experimental group in months 1, 2, 4, 8, 10, 12, and 14. Rats were individually placed in metabolic cages for 10 h to collect urine samples without any treatment (from 8 a.m. to 6 p.m.). After collection, the urine samples were quickly stored at −80 °C. The urine samples were centrifuged at 12,000× *g* for 40 min at 4 °C to remove cell debris. The supernatants were precipitated with three volumes of ethanol at −20 °C overnight and then centrifuged at 12,000× *g* for 30 min. The pellet was resuspended in lysis buffer (8 mol/L urea, 2 mol/L thiourea, 50 mmol/L Tris, and 25 mmol/L DTT). The protein concentration of the urine samples was measured using the Bradford assay.

### 4.3. Measurement of Blood Pressure

A noninvasive blood pressure measuring instrument was used to measure the blood pressure at the tail artery, as previously described [33]. Briefly, rats were fixed on a fixation frame at room temperature until the body temperature increased to 39 °C. The proximal tail was attached to the computer via a dynamic signal acquisition system. The mean BP was obtained from three consecutive readings at 3 min intervals.

### 4.4. Histopathology

At the age of 14 months, 3 rats were subjected to intracardiac perfusion. Whole-body blood was quickly flushed with 0.9% normal saline, and tissues were fixed with 4% paraformaldehyde. The brain and kidney were removed and preserved in 4% paraformaldehyde. Then, the samples were embedded in paraffin, sectioned, and evaluated with hematoxylin and eosin (H&E) staining.

### 4.5. Protein Digestion

One hundred micrograms of urinary proteins from each sample were digested with trypsin (Trypsin Gold, Mass Spec Grade, Promega, Fitchburg, WI, USA) using filter-aided sample preparation (FASP) methods, as previously described [34]. These peptide mixtures were desalted using Oasis HLB cartridges (Waters, Milford, MA, USA) and dried by vacuum evaporation (Thermo Fisher Scientific, Bremen, Germany). The digested peptides (*n* = 36) were redissolved in 0.1% formic acid to a concentration of 0.5 µg/µL, and 1 µg of peptides from each sample was analyzed using LC–MS/MS in DIA mode.

### 4.6. Reverse-Phase Fractionation Spin Column Separation

A pooled sample was generated from equal volumes of digested peptides from each sample. A total of 108 µg of pooled peptides was separated using a high pH reversed-phase peptide fractionation kit (Thermo Pierce, Waltham, MA, USA), according to the manufacturer’s instructions. A step gradient of increasing acetonitrile concentrations (5, 7.5, 10, 12.5, 15, 17.5, 20, and 50% acetonitrile) was added to the columns to elute peptides, and ten different fractions (including the flow-through fraction, wash fraction, and eight step gradient sample fractions) of each sample were collected and dried by vacuum evaporation. The ten fractions were then resuspended in 20 µL of 0.1% formic acid, and 1 µg of peptides from each fraction was used for LC–MS/MS analysis in DDA mode.

### 4.7. LC–MS/MS Analysis

Mass spectrometry acquisition and analysis were performed using an EASY-nLC 1200 chromatography system (Thermo Fisher Scientific, Waltham, MA, USA) and an Orbitrap Fusion Lumos Tribrid mass spectrometer (Thermo Fisher Scientific, Waltham, MA, USA). The iRT reagent (Biognosys, Schlieren, Switzerland) was spiked at a concentration of 1:10 *v*/*v* into all urinary samples for calibration of the retention time of the extracted peptide peaks. The peptide samples were loaded on a trap column (75 µm × 2 cm, 3 µm, C18, 100 Å) and a reverse-phase analysis column (75 µm × 25 cm, 2 µm, C18, 100 Å). The elution gradient was 4–35% buffer B (0.1% formic acid in 80% acetonitrile) at a flow rate of 400 nL/min for 90 min.

One microgram of each fraction from the spin column was analyzed in DDA mode to generate the spectral library. The parameters were set as follows: the full scan was acquired from 350 to 1550 m/z with a resolution of 120,000, and the MS/MS scan was performed with a resolution of 30,000 in Orbitrap; the higher-energy collisional dissociation (HCD) energy was set to 30%; the automatic control (AGC) target was set to 5.0 × 10^4^; and the maximum injection time was set to 45 ms.

One microgram of each sample was analyzed in DIA mode. The variable isolation window of the DIA method with 36 windows was set for DIA. The parameters were set as follows: the full scan was acquired from 350 to 1500 m/z with a resolution of 60,000, the MS/MS scan was acquired from 200 to 2000 m/z with a resolution of 30,000, the HCD energy was set to 32%, the AGC target was set to 1.0 × 10^6^, and the maximum injection time was set to 100 ms.

### 4.8. Data Analysis

The DDA data from the ten fractions were processed using Proteome Discoverer software (version 2.1, Thermo Fisher Scientific, Waltham, MA, USA) and searched against the Swiss-Prot rat database (released in 2017, including 7992 sequences) appended with the iRT peptide sequence. The search parameters were set as follows: two missed trypsin cleavage sites were allowed, the parent ion mass tolerances were set to 10 ppm, the fragment ion mass tolerances were set to 0.02 Da, the carbamidomethyl of cysteine was set as a fixed modification, and the oxidation of methionine was set as a variable modification. The false discovery rate (FDR) of the proteins was less than 1%. A total of 862 protein groups, 4570 peptide groups, and 43,277 peptide spectrum matches were identified. The search results were used to set the variable windows for DIA.

Ten DDA raw files were processed using Spectronaut Pulsar X (Biognosys, Schlieren, Switzerland) with the default parameters to generate the spectral library. Then, 36 raw DIA files for each sample were processed using Spectronaut Pulsar X with the default settings. The results were filtered based on a *q* value cutoff of 0.01. The peptide intensity was based on the peak areas of the respective fragment ions for MS2, and the protein intensity was calculated by summing the intensities of their respective peptides.

### 4.9. Statistical Analysis

The k-nearest neighbor (K-NN) method was used to fill the missing values of protein abundance [35]. As the degree of disease progression may differ, each rat was compared at time points before and after hypertension development to screen for differentially expressed proteins. The differential proteins were screened based on the following criteria: proteins with at least two unique peptides, fold change ≥2 or ≤0.5, and *p* < 0.05 in a two-sided unpaired *t*-test. Group differences resulting in *p* < 0.05 were considered statistically significant.

### 4.10. Functional Annotation of the Differential Proteins

The Database for Annotation, Visualization and Integrated Discovery (DAVID) was used to perform the functional annotation of the differential proteins [36]. The canonical pathways were analyzed with IPA (Ingenuity Systems, Mountain View, CA, USA, v2021) software. All enriched terms had a threshold value of *p* < 0.05.

## 5. Conclusions

This study was a preliminary study with a small sample size. This study demonstrated that changes in hypertension can be reflected in urine proteins. Urinary proteomics has potential in researching the mechanisms underlying hypertension, discovering new drug targets, and developing personalized strategies for antihypertensive treatment.

## Data Availability

Data will be made available on request.

## Supplementary Materials

The following supporting information can be downloaded at: https://www.mdpi.com/article/10.3390/ijms26010021/s1.
