# Supplementary material for: Urinary Proteome Characterization of Stroke-Prone Spontaneously Hypertensive Rats
Source: Int J Mol Sci. 2024 Dec 24;26(1):21. doi: 10.3390/ijms26010021 (PMC11720275; doi:10.3390/ijms26010021)
Supplement: Supplementary file 1 [file ijms-26-00021-s001.zip › ijms-3319522-supplementary.pdf]

**Table S1** Urinary differential proteins of SHRSP model at different time points.

A. Differential proteins identified at different time points in rat 1.

| M4-M1 | | | M8-M4 | | | M14-M12 | | |
|---|---|---|---|---|---|---|---|---|
| Accession | Fold Change | P value | Accession | Fold Change | P value | Accession | Fold Change | P value |
| P97580 | 13.65 | 0.0000 | Q99PS8 | 49.58 | 0.0001 | P18292 | 7.18 | 0.0000 |
| P15399 | 11.37 | 0.0000 | Q07523 | 49.10 | 0.0421 | Q63313 | 6.53 | 0.0001 |
| P12928 | 9.13 | 0.0001 | P01026 | 41.73 | 0.0000 | Q10758 | 6.12 | 0.0005 |
| Q30KJ2 | 8.40 | 0.0048 | P16296 | 37.46 | 0.0026 | P50430 | 5.45 | 0.0114 |
| P04182 | 7.89 | 0.0118 | P04276 | 32.24 | 0.0000 | Q812E9 | 5.33 | 0.0005 |
| Q6TA48 | 7.17 | 0.0008 | Q9WUW3 | 28.29 | 0.0015 | P00697 | 5.00 | 0.0006 |
| P01039 | 6.95 | 0.0040 | P09006 | 27.66 | 0.0000 | P02783 | 4.87 | 0.0075 |
| P22006 | 6.93 | 0.0006 | P12346 | 27.45 | 0.0000 | P01830 | 4.41 | 0.0065 |
| P12346 | 6.15 | 0.0001 | Q62975 | 22.51 | 0.0004 | P15399 | 4.15 | 0.0002 |
| Q00715 | 6.03 | 0.0002 | G3V686 | 21.46 | 0.0022 | Q6IFW6 | 4.10 | 0.0000 |
| P10760 | 5.50 | 0.0001 | Q3KRD8 | 18.19 | 0.0210 | Q6IFV4 | 3.99 | 0.0039 |
| P14141 | 5.44 | 0.0001 | P08934 | 17.64 | 0.0001 | Q6P0K8 | 3.62 | 0.0036 |
| Q6AYS7 | 5.08 | 0.0007 | D3ZTE0 | 16.10 | 0.0007 | Q4FZU2 | 3.41 | 0.0000 |
| P16290 | 4.82 | 0.0001 | P20759 | 16.01 | 0.0002 | Q08415 | 3.23 | 0.0453 |
| Q6AYT0 | 4.66 | 0.0001 | P20761 | 15.65 | 0.0000 | Q6TA48 | 3.19 | 0.0041 |
| P85971 | 4.58 | 0.0003 | P13635 | 15.60 | 0.0024 | Q6IMF3 | 3.13 | 0.0003 |
| O88767 | 4.56 | 0.0057 | P55159 | 15.57 | 0.0009 | Q6IG02 | 3.10 | 0.0003 |
| P55091 | 4.49 | 0.0008 | P25236 | 15.53 | 0.0000 | Q6IFU8 | 3.03 | 0.0000 |
| G3V7W1 | 4.42 | 0.0001 | P01039 | 15.13 | 0.0101 | Q6P6Q2 | 2.89 | 0.0059 |
| P38918 | 4.33 | 0.0001 | Q9QX79 | 14.92 | 0.0000 | P53813 | 2.87 | 0.0010 |
| P00714 | 4.24 | 0.0002 | P55314 | 14.50 | 0.0005 | P97580 | 2.76 | 0.0044 |
| P00774 | 4.17 | 0.0004 | Q9EQV9 | 12.80 | 0.0020 | P05197 | 2.73 | 0.0161 |
| P62815 | 4.02 | 0.0069 | P26644 | 12.12 | 0.0006 | P09656 | 2.68 | 0.0432 |
| Q9R063 | 3.84 | 0.0122 | P17475 | 11.52 | 0.0000 | Q5EGZ1 | 2.62 | 0.0000 |
| Q9WUW8 | 3.79 | 0.0008 | Q62930 | 11.48 | 0.0001 | P11517 | 2.56 | 0.0018 |
| Q9JLJ3 | 3.65 | 0.0005 | Q811M5 | 10.41 | 0.0002 | Q6IG00 | 2.53 | 0.0221 |
| Q5U2Q3 | 3.61 | 0.0005 | Q63416 | 9.34 | 0.0000 | P04642 | 2.53 | 0.0403 |
| P02783 | 3.55 | 0.0062 | Q63678 | 9.30 | 0.0000 | P22006 | 2.49 | 0.0018 |
| Q66HG4 | 3.54 | 0.0014 | P20762 | 8.99 | 0.0005 | P07861 | 2.43 | 0.0001 |
| P62804 | 3.47 | 0.0063 | P02680 | 8.92 | 0.0001 | Q811X6 | 2.38 | 0.0317 |
| P02650 | 3.35 | 0.0002 | P06399 | 8.64 | 0.0000 | Q5FVR3 | 2.32 | 0.0459 |
| P85973 | 3.34 | 0.0014 | P04916 | 8.63 | 0.0002 | P17559 | 2.29 | 0.0023 |
| P04762 | 3.28 | 0.0008 | Q63207 | 8.42 | 0.0011 | Q9WUW8 | 2.25 | 0.0424 |
| Q9EQV6 | 3.24 | 0.0000 | P14480 | 8.28 | 0.0025 | P02091 | 2.20 | 0.0003 |
| P13635 | 3.19 | 0.0001 | P02770 | 7.84 | 0.0036 | P08025 | 2.20 | 0.0067 |
| B0BNN3 | 3.06 | 0.0047 | Q6P734 | 7.02 | 0.0003 | Q920A6 | 0.48 | 0.0100 |

| | | | | | | | | |
|---|---|---|---|---|---|---|---|---|
| P04764 | 3.05 | 0.0008 | P02651 | 6.83 | 0.0007 | P00758 | 0.48 | 0.0001 |
| Q7TQ94 | 2.98 | 0.0001 | P20760 | 6.76 | 0.0027 | P98158 | 0.45 | 0.0006 |
| P11980 | 2.92 | 0.0216 | P01048 | 6.50 | 0.0001 | Q00715 | 0.44 | 0.0016 |
| P05065 | 2.91 | 0.0045 | P08721 | 6.35 | 0.0000 | P27590 | 0.43 | 0.0002 |
| Q06496 | 2.84 | 0.0040 | P08932 | 6.12 | 0.0017 | Q64230 | 0.41 | 0.0005 |
| Q4G075 | 2.71 | 0.0019 | P01805 | 5.94 | 0.0000 | P50123 | 0.41 | 0.0076 |
| P04797 | 2.68 | 0.0002 | P31394 | 5.87 | 0.0001 | Q9JJ19 | 0.41 | 0.0149 |
| P20761 | 2.59 | 0.0004 | Q4QQV8 | 5.86 | 0.0009 | P14740 | 0.40 | 0.0017 |
| Q9R0J8 | 2.58 | 0.0004 | P14046 | 5.20 | 0.0001 | P20611 | 0.39 | 0.0071 |
| O88989 | 2.57 | 0.0039 | P18424 | 5.01 | 0.0029 | P02625 | 0.38 | 0.0021 |
| P25113 | 2.56 | 0.0042 | P35859 | 4.62 | 0.0002 | Q63257 | 0.36 | 0.0298 |
| P07314 | 2.50 | 0.0016 | Q01177 | 4.32 | 0.0011 | P28826 | 0.32 | 0.0110 |
| P20760 | 2.49 | 0.0083 | P36953 | 4.27 | 0.0000 | P07943 | 0.31 | 0.0000 |
| P18757 | 2.48 | 0.0014 | Q62894 | 4.25 | 0.0058 | Q05175 | 0.30 | 0.0068 |
| P04906 | 2.46 | 0.0009 | P02767 | 4.12 | 0.0001 | P04073 | 0.29 | 0.0128 |
| Q63751 | 2.43 | 0.0002 | P06866 | 3.96 | 0.0002 | P19218 | 0.26 | 0.0106 |
| Q812E9 | 2.31 | 0.0395 | Q63621 | 3.92 | 0.0014 | | | |
| Q811X6 | 2.30 | 0.0097 | P53813 | 3.76 | 0.0001 | | | |
| P50430 | 2.29 | 0.0153 | Q64268 | 3.62 | 0.0494 | | | |
| Q920P0 | 2.28 | 0.0111 | P48199 | 3.59 | 0.0036 | | | |
| P06866 | 2.27 | 0.0004 | P60571 | 3.46 | 0.0207 | | | |
| P45479 | 2.26 | 0.0227 | P10959 | 3.45 | 0.0000 | | | |
| P11240 | 2.24 | 0.0030 | P20059 | 3.44 | 0.0001 | | | |
| P09606 | 2.20 | 0.0010 | P31211 | 3.26 | 0.0003 | | | |
| O35244 | 2.17 | 0.0027 | Q03626 | 3.25 | 0.0343 | | | |
| Q9Z339 | 2.11 | 0.0000 | P82450 | 3.21 | 0.0001 | | | |
| P80067 | 2.11 | 0.0117 | P07483 | 3.18 | 0.0472 | | | |
| Q6P6V0 | 2.09 | 0.0114 | P24090 | 3.17 | 0.0000 | | | |
| P98089 | 2.07 | 0.0002 | Q6IE52 | 2.82 | 0.0007 | | | |
| P28494 | 2.05 | 0.0249 | Q9JJS8 | 2.80 | 0.0339 | | | |
| Q6P9T8 | 2.02 | 0.0002 | Q6P0K8 | 2.76 | 0.0008 | | | |
| P23764 | 2.01 | 0.0026 | P14668 | 2.74 | 0.0014 | | | |
| Q9EQX9 | 0.50 | 0.0020 | P04639 | 2.71 | 0.0001 | | | |
| Q6RY07 | 0.49 | 0.0181 | P08649 | 2.71 | 0.0001 | | | |
| iRT-Kit_WR_fusion | 0.49 | 0.0422 | Q6IFV1 | 2.70 | 0.0009 | | | |
| Q02765 | 0.49 | 0.0034 | P55054 | 2.63 | 0.0267 | | | |
| P00762 | 0.48 | 0.0283 | P05545 | 2.56 | 0.0000 | | | |
| Q62632 | 0.48 | 0.0038 | P05544 | 2.55 | 0.0066 | | | |
| P18292 | 0.48 | 0.0007 | P49744 | 2.47 | 0.0409 | | | |

| | | | | | | | | |
|---|---|---|---|---|---|---|---|---|
| P0DP29;P0DP30;P0DP31 | 0.47 | 0.0157 | P01015 | 2.22 | 0.0087 | | | |
| O54861 | 0.47 | 0.0004 | Q30KJ2 | 2.18 | 0.0016 | | | |
| P57097 | 0.47 | 0.0130 | Q63135 | 2.14 | 0.0008 | | | |
| P00787 | 0.47 | 0.0003 | P02764 | 2.06 | 0.0034 | | | |
| P08937 | 0.47 | 0.0039 | P02091 | 2.05 | 0.0001 | | | |
| Q63598 | 0.47 | 0.0006 | Q811X6 | 0.50 | 0.0171 | | | |
| O70535 | 0.47 | 0.0005 | Q6AYE5 | 0.50 | 0.0226 | | | |
| P20766 | 0.45 | 0.0308 | P19468 | 0.50 | 0.0006 | | | |
| O35568 | 0.45 | 0.0001 | P80020 | 0.50 | 0.0029 | | | |
| P00502 | 0.44 | 0.0026 | Q06496 | 0.49 | 0.0000 | | | |
| P30152 | 0.44 | 0.0020 | Q6AXX6 | 0.49 | 0.0003 | | | |
| P02680 | 0.44 | 0.0302 | Q6P6S9 | 0.49 | 0.0099 | | | |
| P04937 | 0.44 | 0.0089 | Q6IRK9 | 0.49 | 0.0016 | | | |
| Q62740 | 0.44 | 0.0011 | P14630 | 0.49 | 0.0032 | | | |
| P11598 | 0.43 | 0.0191 | P04904 | 0.49 | 0.0033 | | | |
| Q8CG45 | 0.43 | 0.0013 | Q64240 | 0.49 | 0.0010 | | | |
| P25031 | 0.42 | 0.0018 | P51635 | 0.48 | 0.0060 | | | |
| Q924B5 | 0.42 | 0.0041 | O35112 | 0.48 | 0.0052 | | | |
| Q810F4 | 0.42 | 0.0005 | P07314 | 0.48 | 0.0036 | | | |
| Q6P6S9 | 0.42 | 0.0017 | P19218 | 0.48 | 0.0014 | | | |
| P18427 | 0.41 | 0.0094 | Q04589 | 0.48 | 0.0009 | | | |
| Q8R4E1 | 0.41 | 0.0403 | P08289 | 0.48 | 0.0028 | | | |
| P30836 | 0.41 | 0.0003 | Q5HZW5 | 0.48 | 0.0064 | | | |
| Q6AXR4 | 0.41 | 0.0018 | Q64604 | 0.48 | 0.0116 | | | |
| P15473 | 0.40 | 0.0011 | O70489 | 0.47 | 0.0021 | | | |
| Q4V885 | 0.39 | 0.0014 | G3V7W1 | 0.47 | 0.0052 | | | |
| Q68FQ2 | 0.39 | 0.0002 | P01681 | 0.47 | 0.0124 | | | |
| Q6AYP5 | 0.39 | 0.0001 | P00787 | 0.47 | 0.0002 | | | |
| P40241 | 0.38 | 0.0005 | P15684 | 0.47 | 0.0010 | | | |
| P60571 | 0.38 | 0.0380 | P52759 | 0.47 | 0.0019 | | | |
| P42123 | 0.38 | 0.0002 | P02783 | 0.46 | 0.0322 | | | |
| P54313 | 0.38 | 0.0269 | Q64573 | 0.46 | 0.0009 | | | |
| Q0PMD2 | 0.37 | 0.0000 | P30121 | 0.46 | 0.0026 | | | |
| P61459 | 0.36 | 0.0007 | P97603 | 0.46 | 0.0084 | | | |
| P07151 | 0.36 | 0.0001 | P27590 | 0.46 | 0.0000 | | | |
| Q63493 | 0.36 | 0.0098 | P18757 | 0.45 | 0.0060 | | | |
| P16310 | 0.36 | 0.0045 | P14562 | 0.45 | 0.0030 | | | |
| Q8JZQ0 | 0.35 | 0.0134 | P01830 | 0.45 | 0.0001 | | | |
| P70490 | 0.35 | 0.0000 | Q6AYD4 | 0.45 | 0.0095 | | | |
| Q3KR97 | 0.35 | 0.0013 | P81827 | 0.45 | 0.0011 | | | |
| Q7TP52 | 0.35 | 0.0057 | O88766 | 0.45 | 0.0006 | | | |

| | | | | | | | | |
|---|---|---|---|---|---|---|---|---|
| Q05820 | 0.34 | 0.0058 | Q08420 | 0.45 | 0.0012 | | | |
| P04073 | 0.34 | 0.0014 | Q9QZQ5 | 0.45 | 0.0047 | | | |
| P18418 | 0.34 | 0.0004 | Q562C9 | 0.45 | 0.0064 | | | |
| P22282 | 0.34 | 0.0012 | Q63257 | 0.44 | 0.0028 | | | |
| Q99PS8 | 0.34 | 0.0010 | P36375 | 0.44 | 0.0099 | | | |
| P02780 | 0.34 | 0.0013 | P30120 | 0.44 | 0.0005 | | | |
| P48037 | 0.34 | 0.0189 | P61972 | 0.44 | 0.0002 | | | |
| P08649 | 0.33 | 0.0029 | Q99MA2 | 0.44 | 0.0061 | | | |
| P20786 | 0.33 | 0.0024 | Q63751 | 0.44 | 0.0010 | | | |
| Q9R0D6 | 0.33 | 0.0108 | P19132 | 0.43 | 0.0100 | | | |
| P15943 | 0.33 | 0.0155 | P23377 | 0.43 | 0.0166 | | | |
| O88917 | 0.33 | 0.0223 | P28826 | 0.43 | 0.0005 | | | |
| P11517 | 0.33 | 0.0099 | P47853 | 0.43 | 0.0035 | | | |
| P62260 | 0.32 | 0.0056 | Q0PMD2 | 0.43 | 0.0002 | | | |
| P07647 | 0.32 | 0.0010 | Q78P75 | 0.43 | 0.0469 | | | |
| P02782 | 0.32 | 0.0018 | P30919 | 0.43 | 0.0000 | | | |
| Q9WVH8 | 0.32 | 0.0032 | O55006 | 0.43 | 0.0004 | | | |
| Q498S8 | 0.32 | 0.0185 | P19804 | 0.42 | 0.0352 | | | |
| Q9JID2 | 0.31 | 0.0239 | Q32KJ6 | 0.42 | 0.0000 | | | |
| P02781 | 0.31 | 0.0006 | Q1WIM1 | 0.42 | 0.0145 | | | |
| P22283 | 0.31 | 0.0282 | Q05695 | 0.42 | 0.0014 | | | |
| P06761 | 0.30 | 0.0016 | P25093 | 0.42 | 0.0010 | | | |
| P47727 | 0.30 | 0.0001 | P55146 | 0.42 | 0.0058 | | | |
| Q811M5 | 0.30 | 0.0368 | P08937 | 0.42 | 0.0026 | | | |
| Q63424 | 0.30 | 0.0049 | Q04807 | 0.42 | 0.0006 | | | |
| P11232 | 0.30 | 0.0029 | P00502 | 0.42 | 0.0004 | | | |
| P35053 | 0.29 | 0.0005 | P15978 | 0.41 | 0.0003 | | | |
| P01830 | 0.29 | 0.0003 | Q10758 | 0.41 | 0.0012 | | | |
| P11348 | 0.29 | 0.0006 | Q9JLJ3 | 0.41 | 0.0023 | | | |
| P07483 | 0.28 | 0.0011 | O70417 | 0.41 | 0.0008 | | | |
| P02692 | 0.28 | 0.0143 | Q8CHN3 | 0.41 | 0.0017 | | | |
| P00731 | 0.28 | 0.0011 | P00786 | 0.41 | 0.0001 | | | |
| P46720 | 0.27 | 0.0007 | O70244 | 0.41 | 0.0000 | | | |
| Q4TU93 | 0.26 | 0.0020 | P21704 | 0.41 | 0.0000 | | | |
| D3ZTE0 | 0.26 | 0.0096 | Q64194 | 0.40 | 0.0021 | | | |
| O55004 | 0.26 | 0.0001 | Q1WIM3 | 0.40 | 0.0009 | | | |
| P26644 | 0.24 | 0.0133 | P47727 | 0.40 | 0.0004 | | | |
| P34080 | 0.24 | 0.0037 | P14740 | 0.40 | 0.0001 | | | |
| Q6GMN2 | 0.23 | 0.0009 | Q68FT5 | 0.40 | 0.0071 | | | |
| Q9QZQ5 | 0.22 | 0.0000 | P98158 | 0.40 | 0.0000 | | | |
| P24090 | 0.20 | 0.0004 | P30710 | 0.39 | 0.0018 | | | |
| P14668 | 0.20 | 0.0000 | P11232 | 0.39 | 0.0000 | | | |
| P35444 | 0.19 | 0.0002 | P19112 | 0.39 | 0.0058 | | | |

| | | | | | | | | |
|---|---|---|---|---|---|---|---|---|
| Q5GRG2 | 0.18 | 0.0001 | Q4QQW8 | 0.39 | 0.0030 | | | |
| P12020 | 0.18 | 0.0001 | P00714 | 0.39 | 0.0015 | | | |
| P06911 | 0.18 | 0.0000 | P26772 | 0.39 | 0.0003 | | | |
| P08723 | 0.17 | 0.0005 | P30152 | 0.39 | 0.0014 | | | |
| P17559 | 0.17 | 0.0004 | P84039 | 0.38 | 0.0442 | | | |
| P01681 | 0.16 | 0.0300 | Q3MIE4 | 0.38 | 0.0238 | | | |
| P07897 | 0.16 | 0.0000 | P00758 | 0.38 | 0.0003 | | | |
| P30710 | 0.15 | 0.0001 | P02761 | 0.38 | 0.0000 | | | |
| P07171 | 0.14 | 0.0000 | Q9JI85 | 0.38 | 0.0012 | | | |
| P30120 | 0.14 | 0.0000 | Q64230 | 0.38 | 0.0002 | | | |
| P42854 | 0.12 | 0.0024 | Q91XN4 | 0.38 | 0.0006 | | | |
| G3V686 | 0.07 | 0.0014 | Q76HN1 | 0.37 | 0.0001 | | | |
| P08689 | 0.07 | 0.0000 | Q63270 | 0.37 | 0.0427 | | | |
| P55054 | 0.02 | 0.0006 | Q03336 | 0.37 | 0.0069 | | | |
| | | | Q499T2 | 0.37 | 0.0001 | | | |
| | | | P50430 | 0.37 | 0.0084 | | | |
| | | | Q99J86 | 0.37 | 0.0003 | | | |
| | | | B0BND0 | 0.37 | 0.0006 | | | |
| | | | P04785 | 0.37 | 0.0008 | | | |
| | | | Q9EQV6 | 0.37 | 0.0005 | | | |
| | | | Q6IG00 | 0.37 | 0.0083 | | | |
| | | | Q62632 | 0.37 | 0.0025 | | | |
| | | | P11598 | 0.36 | 0.0115 | | | |
| | | | P10758 | 0.36 | 0.0010 | | | |
| | | | Q9Z1Y3 | 0.36 | 0.0004 | | | |
| | | | P07522 | 0.36 | 0.0007 | | | |
| | | | P38983 | 0.36 | 0.0005 | | | |
| | | | P63018 | 0.36 | 0.0010 | | | |
| | | | Q66H12 | 0.36 | 0.0038 | | | |
| | | | P19939 | 0.36 | 0.0298 | | | |
| | | | P52796 | 0.36 | 0.0009 | | | |
| | | | Q00657 | 0.36 | 0.0015 | | | |
| | | | P70490 | 0.36 | 0.0001 | | | |
| | | | P63102 | 0.36 | 0.0019 | | | |
| | | | Q9WTQ2 | 0.35 | 0.0000 | | | |
| | | | Q5FVH2 | 0.35 | 0.0007 | | | |
| | | | P22734 | 0.35 | 0.0055 | | | |
| | | | O35568 | 0.35 | 0.0000 | | | |
| | | | P05065 | 0.35 | 0.0031 | | | |
| | | | P13852 | 0.35 | 0.0001 | | | |
| | | | Q63475 | 0.35 | 0.0076 | | | |
| | | | Q62867 | 0.34 | 0.0011 | | | |
| | | | P43303 | 0.34 | 0.0001 | | | |

| | | | | | | | | |
|---|---|---|---|---|---|---|---|---|
| | | | P08460 | 0.34 | 0.0001 | | | |
| | | | Q68FS4 | 0.34 | 0.0026 | | | |
| | | | Q68FP1 | 0.34 | 0.0000 | | | |
| | | | Q6P7S1 | 0.34 | 0.0003 | | | |
| | | | Q6IUU3 | 0.34 | 0.0072 | | | |
| | | | Q8R5M3 | 0.34 | 0.0104 | | | |
| | | | P07150 | 0.34 | 0.0064 | | | |
| | | | Q02974 | 0.33 | 0.0073 | | | |
| | | | P07897 | 0.33 | 0.0021 | | | |
| | | | P80067 | 0.33 | 0.0066 | | | |
| | | | P36373 | 0.33 | 0.0013 | | | |
| | | | Q568Z6 | 0.33 | 0.0001 | | | |
| | | | Q68FQ2 | 0.33 | 0.0010 | | | |
| | | | P04642 | 0.33 | 0.0001 | | | |
| | | | P50137 | 0.32 | 0.0184 | | | |
| | | | Q63493 | 0.32 | 0.0087 | | | |
| | | | Q80WY6 | 0.32 | 0.0256 | | | |
| | | | Q9QWJ9 | 0.32 | 0.0105 | | | |
| | | | Q8R2H5 | 0.32 | 0.0364 | | | |
| | | | P0CG51 | 0.32 | 0.0002 | | | |
| | | | Q9JIK1 | 0.31 | 0.0021 | | | |
| | | | P11980 | 0.31 | 0.0014 | | | |
| | | | Q63474 | 0.31 | 0.0006 | | | |
| | | | P22057 | 0.31 | 0.0000 | | | |
| | | | Q08464 | 0.31 | 0.0000 | | | |
| | | | Q5U2Q3 | 0.30 | 0.0005 | | | |
| | | | Q63083 | 0.30 | 0.0000 | | | |
| | | | P61589 | 0.30 | 0.0022 | | | |
| | | | Q6AYT0 | 0.30 | 0.0007 | | | |
| | | | P97675 | 0.29 | 0.0029 | | | |
| | | | Q05820 | 0.29 | 0.0004 | | | |
| | | | Q5XI43 | 0.29 | 0.0159 | | | |
| | | | Q5FVR3 | 0.29 | 0.0039 | | | |
| | | | P21581 | 0.29 | 0.0003 | | | |
| | | | P97546 | 0.28 | 0.0005 | | | |
| | | | Q66HG4 | 0.28 | 0.0021 | | | |
| | | | Q9Z2Y9 | 0.28 | 0.0111 | | | |
| | | | Q6AXR4 | 0.28 | 0.0000 | | | |
| | | | P16290 | 0.28 | 0.0018 | | | |
| | | | P15083 | 0.28 | 0.0002 | | | |
| | | | P02650 | 0.28 | 0.0002 | | | |
| | | | P46953 | 0.28 | 0.0000 | | | |
| | | | P29288 | 0.28 | 0.0001 | | | |

| | | | | | | | | |
|---|---|---|---|---|---|---|---|---|
| | | | P24594 | 0.28 | 0.0004 | | | |
| | | | P83121 | 0.28 | 0.0012 | | | |
| | | | P53369 | 0.28 | 0.0004 | | | |
| | | | Q4FZV0 | 0.27 | 0.0007 | | | |
| | | | Q8VIF7 | 0.27 | 0.0049 | | | |
| | | | P46462 | 0.27 | 0.0187 | | | |
| | | | Q9ESG3 | 0.27 | 0.0014 | | | |
| | | | P27867 | 0.27 | 0.0013 | | | |
| | | | P68255 | 0.27 | 0.0009 | | | |
| | | | Q920A6 | 0.27 | 0.0004 | | | |
| | | | Q5FVR0 | 0.27 | 0.0266 | | | |
| | | | Q4V885 | 0.26 | 0.0021 | | | |
| | | | P04762 | 0.26 | 0.0007 | | | |
| | | | P97710 | 0.26 | 0.0000 | | | |
| | | | Q6P767 | 0.26 | 0.0004 | | | |
| | | | P42854 | 0.26 | 0.0000 | | | |
| | | | P04906 | 0.25 | 0.0003 | | | |
| | | | P04937 | 0.25 | 0.0013 | | | |
| | | | P80202 | 0.25 | 0.0010 | | | |
| | | | Q6P7A9 | 0.25 | 0.0000 | | | |
| | | | P08010 | 0.24 | 0.0229 | | | |
| | | | P27274 | 0.24 | 0.0000 | | | |
| | | | P62898 | 0.24 | 0.0002 | | | |
| | | | O88767 | 0.24 | 0.0061 | | | |
| | | | P09606 | 0.23 | 0.0020 | | | |
| | | | Q641X3 | 0.23 | 0.0003 | | | |
| | | | Q924B5 | 0.22 | 0.0048 | | | |
| | | | Q9JLS4 | 0.22 | 0.0019 | | | |
| | | | P19629 | 0.22 | 0.0035 | | | |
| | | | Q9R0T4 | 0.22 | 0.0009 | | | |
| | | | P04764 | 0.22 | 0.0004 | | | |
| | | | Q7M0E3 | 0.22 | 0.0125 | | | |
| | | | P05964 | 0.22 | 0.0088 | | | |
| | | | P70709 | 0.22 | 0.0001 | | | |
| | | | B5DFC9 | 0.21 | 0.0015 | | | |
| | | | Q9ES87 | 0.21 | 0.0000 | | | |
| | | | Q9EPB1 | 0.21 | 0.0000 | | | |
| | | | Q68FR8 | 0.21 | 0.0018 | | | |
| | | | P55091 | 0.21 | 0.0006 | | | |
| | | | P02625 | 0.20 | 0.0007 | | | |
| | | | P04182 | 0.20 | 0.0163 | | | |
| | | | P41562 | 0.20 | 0.0004 | | | |
| | | | Q9QZK9 | 0.19 | 0.0004 | | | |

| | | | | | | | | |
|---|---|---|---|---|---|---|---|---|
| | | | Q63279 | 0.19 | 0.0007 | | | |
| | | | Q8CFN2 | 0.19 | 0.0001 | | | |
| | | | P12020 | 0.19 | 0.0054 | | | |
| | | | P00774 | 0.19 | 0.0002 | | | |
| | | | P00689 | 0.18 | 0.0000 | | | |
| | | | Q00238 | 0.18 | 0.0035 | | | |
| | | | P07647 | 0.18 | 0.0000 | | | |
| | | | Q9JHY1 | 0.17 | 0.0001 | | | |
| | | | Q6TA48 | 0.17 | 0.0004 | | | |
| | | | P02781 | 0.17 | 0.0030 | | | |
| | | | P02782 | 0.16 | 0.0006 | | | |
| | | | P29598 | 0.16 | 0.0000 | | | |
| | | | Q8K1G0 | 0.16 | 0.0001 | | | |
| | | | P50399 | 0.16 | 0.0032 | | | |
| | | | P68370 | 0.16 | 0.0010 | | | |
| | | | P08592 | 0.15 | 0.0004 | | | |
| | | | P45479 | 0.15 | 0.0052 | | | |
| | | | P20611 | 0.14 | 0.0000 | | | |
| | | | P17164 | 0.13 | 0.0162 | | | |
| | | | P36374 | 0.12 | 0.0000 | | | |
| | | | P22283 | 0.12 | 0.0005 | | | |
| | | | P02780 | 0.12 | 0.0003 | | | |
| | | | Q9R0J8 | 0.12 | 0.0000 | | | |
| | | | Q5GRG2 | 0.11 | 0.0013 | | | |
| | | | P31044 | 0.11 | 0.0003 | | | |
| | | | Q63772 | 0.11 | 0.0150 | | | |
| | | | Q6P9T8 | 0.11 | 0.0000 | | | |
| | | | Q711G3 | 0.10 | 0.0025 | | | |
| | | | Q6RUV5 | 0.10 | 0.0001 | | | |
| | | | P22282 | 0.09 | 0.0010 | | | |
| | | | D3ZHA0 | 0.08 | 0.0069 | | | |
| | | | P06911 | 0.05 | 0.0012 | | | |
| | | | Q8VI04 | 0.03 | 0.0005 | | | |

B. Differential proteins identified at different time points in rat 2.

| **M4-M1** | | | **M8-M4** | | | **M14-M12** | | |
|---|---|---|---|---|---|---|---|---|
| **Accession** | **Fold Change** | **P value** | **Accession** | **Fold Change** | **P value** | **Accession** | **Fold Change** | **P value** |
| P36374 | 18.96 | 0.0000 | Q07523 | 41.63 | 0.0284 | Q6P6Q2 | 7.99 | 0.0175 |
| P22006 | 18.16 | 0.0003 | Q99PS8 | 31.86 | 0.0000 | Q6IMF3 | 6.01 | 0.0000 |
| P20761 | 11.13 | 0.0001 | P01026 | 30.58 | 0.0000 | Q6IFU8 | 5.98 | 0.0000 |
| P19939 | 8.02 | 0.0007 | P55159 | 24.55 | 0.0003 | Q6IFW6 | 5.61 | 0.0001 |

| | | | | | | | | |
|---|---|---|---|---|---|---|---|---|
| P20760 | 7.60 | 0.0000 | P09006 | 17.06 | 0.0000 | P08723 | 5.54 | 0.0001 |
| P14141 | 6.09 | 0.0000 | Q9WUW3 | 16.75 | 0.0015 | Q4FZU2 | 5.12 | 0.0002 |
| Q02401 | 5.62 | 0.0304 | P12346 | 14.42 | 0.0000 | Q6MG71 | 4.67 | 0.0004 |
| P07647 | 5.55 | 0.0003 | P02091 | 14.17 | 0.0000 | P14562 | 4.55 | 0.0425 |
| P27590 | 5.12 | 0.0001 | P13635 | 13.34 | 0.0004 | Q6IFV4 | 4.13 | 0.0032 |
| P02781 | 5.03 | 0.0002 | P55314 | 12.30 | 0.0047 | Q10758 | 4.02 | 0.0280 |
| P06866 | 4.80 | 0.0003 | P20762 | 12.13 | 0.0000 | P07861 | 3.85 | 0.0000 |
| Q63772 | 4.57 | 0.0051 | P04276 | 11.61 | 0.0000 | Q6IG00 | 3.83 | 0.0116 |
| P02780 | 4.25 | 0.0000 | P02651 | 11.08 | 0.0000 | P02781 | 3.72 | 0.0005 |
| P22282 | 3.98 | 0.0000 | P25236 | 10.71 | 0.0002 | Q6IG02 | 3.70 | 0.0000 |
| P12346 | 3.96 | 0.0000 | P20759 | 10.62 | 0.0000 | G3V7W1 | 3.65 | 0.0048 |
| P02782 | 3.82 | 0.0006 | P08934 | 9.94 | 0.0001 | Q63751 | 3.61 | 0.0082 |
| P04639 | 3.78 | 0.0004 | P20761 | 8.86 | 0.0000 | P06760 | 3.58 | 0.0011 |
| Q6P0K8 | 3.71 | 0.0000 | P17475 | 8.36 | 0.0000 | P02625 | 3.57 | 0.0012 |
| Q03191 | 3.71 | 0.0003 | Q63416 | 8.23 | 0.0000 | P00697 | 3.43 | 0.0001 |
| Q8VI04 | 3.67 | 0.0091 | Q62930 | 7.98 | 0.0001 | P08937 | 3.39 | 0.0002 |
| Q68FR6 | 3.60 | 0.0051 | Q4QQV8 | 7.86 | 0.0000 | P18292 | 3.25 | 0.0000 |
| P02625 | 3.58 | 0.0001 | P20760 | 7.57 | 0.0000 | Q62740 | 3.17 | 0.0177 |
| Q9R063 | 3.49 | 0.0045 | P01946 | 7.29 | 0.0002 | P02780 | 3.16 | 0.0006 |
| Q63678 | 3.46 | 0.0001 | P04916 | 6.86 | 0.0008 | P36374 | 3.01 | 0.0005 |
| Q9WUC4 | 3.35 | 0.0058 | Q6P734 | 6.58 | 0.0002 | Q6IFU7 | 2.94 | 0.0176 |
| P19218 | 3.33 | 0.0000 | Q9QX79 | 6.47 | 0.0005 | P53813 | 2.92 | 0.0061 |
| P02764 | 3.33 | 0.0005 | Q62975 | 6.10 | 0.0130 | Q63598 | 2.91 | 0.0011 |
| P02770 | 3.23 | 0.0023 | P04639 | 5.90 | 0.0000 | P00714 | 2.80 | 0.0005 |
| Q99PW3 | 3.04 | 0.0087 | P01048 | 5.88 | 0.0000 | P10960 | 2.78 | 0.0213 |
| P83121 | 3.00 | 0.0004 | Q9EQV9 | 5.86 | 0.0107 | Q05820 | 2.60 | 0.0289 |
| P17475 | 2.98 | 0.0003 | P31394 | 5.66 | 0.0028 | P02782 | 2.59 | 0.0389 |
| P20788 | 2.96 | 0.0240 | Q63678 | 5.58 | 0.0000 | Q9R063 | 2.56 | 0.0221 |
| Q62638 | 2.94 | 0.0003 | P08932 | 5.58 | 0.0001 | P22282 | 2.54 | 0.0003 |
| Q6IFU7 | 2.94 | 0.0007 | P02770 | 5.47 | 0.0017 | Q4QQV8 | 2.51 | 0.0144 |
| P21744 | 2.93 | 0.0387 | P11517 | 4.96 | 0.0025 | Q6AYE5 | 2.41 | 0.0001 |
| P55054 | 2.91 | 0.0018 | P14046 | 4.96 | 0.0000 | P14841 | 2.34 | 0.0019 |
| Q63313 | 2.87 | 0.0002 | P02767 | 4.70 | 0.0001 | B0BND0 | 2.31 | 0.0073 |
| P13635 | 2.85 | 0.0015 | P06399 | 4.46 | 0.0002 | P07647 | 2.30 | 0.0001 |
| P24594 | 2.82 | 0.0001 | P14668 | 4.38 | 0.0307 | Q63313 | 2.19 | 0.0051 |
| P10354 | 2.81 | 0.0021 | P48199 | 4.22 | 0.0002 | P35577 | 2.18 | 0.0030 |
| P23764 | 2.72 | 0.0040 | Q6IE52 | 3.88 | 0.0001 | Q9WVH8 | 2.15 | 0.0053 |
| P22283 | 2.66 | 0.0010 | Q9JID2 | 3.71 | 0.0067 | P49744 | 2.14 | 0.0485 |
| P00714 | 2.66 | 0.0176 | P02680 | 3.62 | 0.0001 | B0BNN3 | 2.05 | 0.0095 |
| Q9Z1Y3 | 2.63 | 0.0000 | Q03626 | 3.55 | 0.0065 | Q07936 | 2.05 | 0.0406 |
| P19132 | 2.59 | 0.0322 | P53790 | 3.53 | 0.0102 | Q32KJ6 | 2.03 | 0.0398 |
| P81827 | 2.59 | 0.0001 | P14480 | 3.49 | 0.0000 | P15083 | 2.03 | 0.0191 |
| P05371 | 2.59 | 0.0020 | D3ZTE0 | 3.43 | 0.0088 | P30152 | 2.02 | 0.0000 |

| | | | | | | | | |
|---|---|---|---|---|---|---|---|---|
| Q3KR97 | 2.58 | 0.0100 | P36953 | 3.28 | 0.0007 | P02767 | 2.01 | 0.0001 |
| P04276 | 2.58 | 0.0006 | P31211 | 3.25 | 0.0000 | P05544 | 0.50 | 0.0014 |
| Q920A6 | 2.57 | 0.0004 | P53813 | 3.18 | 0.0004 | Q6P6V0 | 0.46 | 0.0019 |
| Q1WIM3 | 2.57 | 0.0014 | Q811M5 | 3.16 | 0.0023 | Q64319 | 0.45 | 0.0417 |
| P35467 | 2.48 | 0.0013 | P07483 | 3.07 | 0.0196 | Q9QZ76 | 0.44 | 0.0035 |
| Q5XIE8 | 2.48 | 0.0081 | P26644 | 2.99 | 0.0000 | P07943 | 0.44 | 0.0068 |
| P15083 | 2.45 | 0.0004 | Q63621 | 2.96 | 0.0000 | Q00238 | 0.44 | 0.0359 |
| Q6P6Q2 | 2.45 | 0.0001 | Q63617 | 2.94 | 0.0069 | P00774 | 0.42 | 0.0007 |
| P36375 | 2.44 | 0.0044 | P08650 | 2.76 | 0.0273 | P80254 | 0.40 | 0.0281 |
| P80202 | 2.38 | 0.0006 | Q5RKI0 | 2.61 | 0.0229 | Q03191 | 0.39 | 0.0287 |
| P01048 | 2.34 | 0.0016 | P06911 | 2.60 | 0.0002 | P11980 | 0.36 | 0.0315 |
| P09006 | 2.34 | 0.0023 | Q63424 | 2.59 | 0.0000 | Q6P9T8 | 0.34 | 0.0187 |
| P02767 | 2.34 | 0.0003 | P82450 | 2.48 | 0.0006 | P15399 | 0.28 | 0.0019 |
| P55281 | 2.33 | 0.0075 | Q01177 | 2.47 | 0.0001 | P11240 | 0.23 | 0.0007 |
| P61972 | 2.29 | 0.0001 | P10959 | 2.39 | 0.0008 | Q63772 | 0.21 | 0.0106 |
| P70709 | 2.26 | 0.0189 | P05544 | 2.25 | 0.0001 | Q62638 | 0.19 | 0.0302 |
| Q9R0J8 | 2.24 | 0.0146 | P06866 | 2.19 | 0.0002 | Q30KJ2 | 0.18 | 0.0057 |
| B0BNN3 | 2.19 | 0.0254 | Q9ESV6 | 2.16 | 0.0301 | P97580 | 0.11 | 0.0000 |
| Q6TMA8 | 2.15 | 0.0037 | Q9WTW7 | 2.14 | 0.0046 | | | |
| P00786 | 2.14 | 0.0005 | P01015 | 2.12 | 0.0005 | | | |
| P14046 | 2.14 | 0.0007 | Q63135 | 2.08 | 0.0001 | | | |
| P05964 | 2.13 | 0.0362 | P04642 | 2.07 | 0.0016 | | | |
| Q4G075 | 2.13 | 0.0202 | P98089 | 2.07 | 0.0144 | | | |
| P80067 | 2.11 | 0.0085 | Q4KLZ6 | 2.03 | 0.0270 | | | |
| P08934 | 2.10 | 0.0014 | O54861 | 0.50 | 0.0181 | | | |
| Q9QX79 | 2.10 | 0.0002 | A9UMV8 | 0.50 | 0.0050 | | | |
| P05545 | 2.06 | 0.0006 | P47820 | 0.50 | 0.0342 | | | |
| Q62930 | 2.05 | 0.0039 | Q01460 | 0.50 | 0.0003 | | | |
| O35078 | 2.04 | 0.0364 | Q6AYE5 | 0.50 | 0.0125 | | | |
| P80020 | 2.02 | 0.0029 | D3Z9M3 | 0.50 | 0.0399 | | | |
| Q4QQV8 | 2.02 | 0.0040 | P02780 | 0.49 | 0.0001 | | | |
| Q63135 | 2.02 | 0.0000 | Q63313 | 0.49 | 0.0062 | | | |
| Q63083 | 2.01 | 0.0002 | P55146 | 0.49 | 0.0096 | | | |
| P55091 | 2.00 | 0.0036 | P18418 | 0.49 | 0.0027 | | | |
| Q8CJD3 | 2.00 | 0.0008 | Q63716 | 0.48 | 0.0124 | | | |
| P27139 | 0.49 | 0.0000 | P22734 | 0.48 | 0.0252 | | | |
| P61206 | 0.48 | 0.0144 | D3ZHA0 | 0.48 | 0.0209 | | | |
| Q9QZQ5 | 0.48 | 0.0076 | Q9EQS0 | 0.47 | 0.0055 | | | |
| P62260 | 0.47 | 0.0110 | P00787 | 0.47 | 0.0112 | | | |
| Q63751 | 0.47 | 0.0076 | Q8CFN2 | 0.47 | 0.0319 | | | |
| P15684 | 0.47 | 0.0001 | Q03336 | 0.47 | 0.0005 | | | |
| Q68FQ2 | 0.46 | 0.0033 | Q63257 | 0.47 | 0.0002 | | | |
| Q920P0 | 0.46 | 0.0016 | P30904 | 0.46 | 0.0478 | | | |

| | | | | | | | | |
|---|---|---|---|---|---|---|---|---|
| P57097 | 0.46 | 0.0145 | P98158 | 0.46 | 0.0000 | | | |
| Q62740 | 0.46 | 0.0038 | P15978 | 0.46 | 0.0003 | | | |
| Q63279 | 0.46 | 0.0025 | P23680 | 0.45 | 0.0063 | | | |
| P97574 | 0.46 | 0.0035 | Q6IFW6 | 0.45 | 0.0001 | | | |
| P28037 | 0.45 | 0.0074 | P13596 | 0.45 | 0.0006 | | | |
| Q6MG61 | 0.45 | 0.0009 | Q642A7 | 0.44 | 0.0033 | | | |
| Q9QZA2 | 0.45 | 0.0087 | O35568 | 0.44 | 0.0120 | | | |
| P51907 | 0.45 | 0.0037 | P13832;P18666 | 0.44 | 0.0295 | | | |
| P25113 | 0.44 | 0.0003 | Q6IRK9 | 0.44 | 0.0116 | | | |
| Q63416 | 0.44 | 0.0476 | Q08406 | 0.44 | 0.0461 | | | |
| P48037 | 0.44 | 0.0007 | Q924B5 | 0.43 | 0.0151 | | | |
| Q8JZQ0 | 0.43 | 0.0032 | Q99J86 | 0.43 | 0.0041 | | | |
| O70377 | 0.43 | 0.0309 | P21704 | 0.43 | 0.0006 | | | |
| P16290 | 0.43 | 0.0022 | B2RYW9 | 0.43 | 0.0006 | | | |
| P07483 | 0.43 | 0.0040 | Q6IG00 | 0.43 | 0.0024 | | | |
| Q9Z0V6 | 0.43 | 0.0098 | P11232 | 0.43 | 0.0059 | | | |
| P60711 | 0.43 | 0.0052 | P07150 | 0.43 | 0.0058 | | | |
| D4A1J4 | 0.42 | 0.0028 | P07647 | 0.43 | 0.0012 | | | |
| Q80W57 | 0.41 | 0.0281 | P35053 | 0.43 | 0.0023 | | | |
| P10719 | 0.41 | 0.0042 | Q64604 | 0.43 | 0.0173 | | | |
| Q68FR8 | 0.41 | 0.0014 | Q810F4 | 0.43 | 0.0241 | | | |
| P07861 | 0.41 | 0.0000 | P07154 | 0.42 | 0.0083 | | | |
| Q6AXS4 | 0.41 | 0.0063 | P11030 | 0.42 | 0.0017 | | | |
| Q6P7S1 | 0.40 | 0.0002 | P29288 | 0.42 | 0.0148 | | | |
| Q9JID2 | 0.40 | 0.0113 | Q6AYD4 | 0.41 | 0.0254 | | | |
| O70244 | 0.40 | 0.0040 | Q9Z339 | 0.41 | 0.0003 | | | |
| P85973 | 0.39 | 0.0098 | P38983 | 0.41 | 0.0066 | | | |
| P31430 | 0.39 | 0.0322 | P15473 | 0.41 | 0.0000 | | | |
| P29975 | 0.39 | 0.0039 | Q7TPA5 | 0.41 | 0.0031 | | | |
| P50123 | 0.39 | 0.0001 | P27590 | 0.41 | 0.0005 | | | |
| P48500 | 0.39 | 0.0013 | Q6P0K8 | 0.40 | 0.0021 | | | |
| Q9QWJ9 | 0.39 | 0.0122 | Q04589 | 0.40 | 0.0017 | | | |
| P05197 | 0.39 | 0.0032 | Q7TP52 | 0.40 | 0.0171 | | | |
| Q9Z0W7 | 0.38 | 0.0001 | P51635 | 0.40 | 0.0006 | | | |
| P00507 | 0.37 | 0.0102 | O35078 | 0.40 | 0.0217 | | | |
| P13221 | 0.37 | 0.0005 | P14841 | 0.40 | 0.0045 | | | |
| P11348 | 0.37 | 0.0081 | Q9EPF2 | 0.40 | 0.0383 | | | |
| P47853 | 0.37 | 0.0015 | Q64230 | 0.40 | 0.0000 | | | |
| Q3KRC4 | 0.36 | 0.0001 | P55091 | 0.40 | 0.0075 | | | |
| P00502 | 0.36 | 0.0003 | Q6AYP5 | 0.40 | 0.0025 | | | |
| P08649 | 0.36 | 0.0022 | P04785 | 0.40 | 0.0002 | | | |
| P51635 | 0.36 | 0.0001 | P46953 | 0.40 | 0.0009 | | | |

| | | | | | | | | |
|---|---|---|---|---|---|---|---|---|
| Q9QXQ0 | 0.36 | 0.0003 | P36373 | 0.39 | 0.0030 | | | |
| Q641Z6 | 0.35 | 0.0002 | P26772 | 0.39 | 0.0017 | | | |
| Q08415 | 0.35 | 0.0076 | P02650 | 0.39 | 0.0003 | | | |
| B5DFC9 | 0.34 | 0.0009 | P23377 | 0.39 | 0.0383 | | | |
| Q9WUW8 | 0.34 | 0.0041 | P20786 | 0.39 | 0.0005 | | | |
| P24090 | 0.32 | 0.0000 | P24368 | 0.38 | 0.0094 | | | |
| Q62687 | 0.32 | 0.0058 | P02761 | 0.38 | 0.0001 | | | |
| P06685 | 0.32 | 0.0041 | P0DMW0 | 0.37 | 0.0000 | | | |
| Q9JJ40 | 0.32 | 0.0008 | P80254 | 0.37 | 0.0036 | | | |
| P15399 | 0.31 | 0.0002 | Q6AYS7 | 0.37 | 0.0003 | | | |
| Q9QYU4 | 0.31 | 0.0011 | Q9EQX9 | 0.37 | 0.0013 | | | |
| Q99MA2 | 0.30 | 0.0001 | P11598 | 0.37 | 0.0268 | | | |
| P51647 | 0.29 | 0.0013 | P10252 | 0.37 | 0.0002 | | | |
| Q0PMD2 | 0.28 | 0.0001 | Q675A5 | 0.37 | 0.0003 | | | |
| P02091 | 0.28 | 0.0013 | Q6AXR4 | 0.37 | 0.0009 | | | |
| Q64602 | 0.27 | 0.0002 | Q9R0T4 | 0.36 | 0.0003 | | | |
| Q812E9 | 0.27 | 0.0008 | Q62867 | 0.36 | 0.0006 | | | |
| P00731 | 0.27 | 0.0004 | P80020 | 0.36 | 0.0022 | | | |
| Q06496 | 0.27 | 0.0007 | Q6P6R2 | 0.36 | 0.0469 | | | |
| P97580 | 0.27 | 0.0008 | P85971 | 0.36 | 0.0000 | | | |
| Q63598 | 0.26 | 0.0004 | P19804 | 0.36 | 0.0001 | | | |
| Q5RKI7 | 0.26 | 0.0080 | P08937 | 0.35 | 0.0001 | | | |
| P35444 | 0.25 | 0.0003 | P07943 | 0.35 | 0.0004 | | | |
| P04073 | 0.25 | 0.0008 | P23785 | 0.35 | 0.0001 | | | |
| P55260 | 0.24 | 0.0028 | P36374 | 0.35 | 0.0000 | | | |
| P07171 | 0.24 | 0.0013 | Q68FS4 | 0.35 | 0.0379 | | | |
| P53790 | 0.24 | 0.0179 | P19218 | 0.35 | 0.0008 | | | |
| Q6P6S9 | 0.23 | 0.0001 | P06760 | 0.35 | 0.0095 | | | |
| P43427 | 0.22 | 0.0012 | P04218 | 0.35 | 0.0037 | | | |
| Q64319 | 0.22 | 0.0000 | Q9JI85 | 0.35 | 0.0449 | | | |
| P47727 | 0.21 | 0.0004 | P30121 | 0.35 | 0.0055 | | | |
| P07897 | 0.20 | 0.0002 | P61972 | 0.34 | 0.0000 | | | |
| P13596 | 0.20 | 0.0014 | P30120 | 0.34 | 0.0018 | | | |
| P08426 | 0.20 | 0.0156 | P04764 | 0.34 | 0.0002 | | | |
| P17559 | 0.18 | 0.0001 | O88766 | 0.34 | 0.0000 | | | |
| Q9WTW7 | 0.18 | 0.0005 | Q63475 | 0.34 | 0.0016 | | | |
| Q63424 | 0.17 | 0.0001 | P22282 | 0.34 | 0.0001 | | | |
| P42854 | 0.15 | 0.0002 | O70417 | 0.34 | 0.0017 | | | |
| P46720 | 0.15 | 0.0023 | Q76HN1 | 0.34 | 0.0015 | | | |
| P04642 | 0.13 | 0.0000 | P23764 | 0.33 | 0.0031 | | | |
| P42123 | 0.11 | 0.0000 | P97710 | 0.33 | 0.0004 | | | |
| P14668 | 0.10 | 0.0000 | P15943 | 0.32 | 0.0031 | | | |
| Q99041 | 0.10 | 0.0000 | Q00657 | 0.32 | 0.0015 | | | |

| | | | | | | | | |
|---|---|---|---|---|---|---|---|---|
| Q4KLZ6 | 0.10 | 0.0006 | P81827 | 0.32 | 0.0001 | | | |
| | | | Q68FQ2 | 0.32 | 0.0225 | | | |
| | | | P01830 | 0.32 | 0.0009 | | | |
| | | | Q8R5M3 | 0.32 | 0.0025 | | | |
| | | | P07522 | 0.32 | 0.0016 | | | |
| | | | Q1WIM1 | 0.32 | 0.0025 | | | |
| | | | Q63467 | 0.32 | 0.0000 | | | |
| | | | Q641X3 | 0.32 | 0.0011 | | | |
| | | | Q6RY07 | 0.31 | 0.0048 | | | |
| | | | Q9WUC4 | 0.31 | 0.0062 | | | |
| | | | P36375 | 0.31 | 0.0051 | | | |
| | | | P19814 | 0.31 | 0.0020 | | | |
| | | | P53369 | 0.31 | 0.0006 | | | |
| | | | Q64240 | 0.31 | 0.0005 | | | |
| | | | Q8VIF7 | 0.31 | 0.0005 | | | |
| | | | P17046 | 0.31 | 0.0010 | | | |
| | | | P30152 | 0.30 | 0.0001 | | | |
| | | | P52759 | 0.30 | 0.0001 | | | |
| | | | P17164 | 0.30 | 0.0178 | | | |
| | | | P97603 | 0.30 | 0.0005 | | | |
| | | | P41562 | 0.30 | 0.0000 | | | |
| | | | Q5FVH2 | 0.30 | 0.0005 | | | |
| | | | Q9R0D6 | 0.30 | 0.0035 | | | |
| | | | O70489 | 0.30 | 0.0001 | | | |
| | | | Q9WTQ2 | 0.30 | 0.0105 | | | |
| | | | Q08420 | 0.30 | 0.0011 | | | |
| | | | Q32KJ6 | 0.29 | 0.0113 | | | |
| | | | Q9JIK1 | 0.29 | 0.0001 | | | |
| | | | P61459 | 0.29 | 0.0015 | | | |
| | | | P15399 | 0.29 | 0.0004 | | | |
| | | | O55004 | 0.29 | 0.0001 | | | |
| | | | Q62740 | 0.29 | 0.0071 | | | |
| | | | P08025 | 0.29 | 0.0001 | | | |
| | | | P02692 | 0.29 | 0.0119 | | | |
| | | | P62898 | 0.29 | 0.0003 | | | |
| | | | P08460 | 0.29 | 0.0000 | | | |
| | | | Q9EPB1 | 0.28 | 0.0008 | | | |
| | | | P24594 | 0.28 | 0.0000 | | | |
| | | | Q711G3 | 0.28 | 0.0008 | | | |
| | | | Q5U2Q3 | 0.28 | 0.0003 | | | |
| | | | P97546 | 0.28 | 0.0013 | | | |
| | | | P15083 | 0.28 | 0.0002 | | | |
| | | | Q10758 | 0.28 | 0.0002 | | | |

| | | | | | | | | |
|---|---|---|---|---|---|---|---|---|
| | | | Q63474 | 0.28 | 0.0067 | | | |
| | | | O35112 | 0.27 | 0.0015 | | | |
| | | | P43303 | 0.27 | 0.0000 | | | |
| | | | P97605 | 0.27 | 0.0096 | | | |
| | | | Q9JLS4 | 0.27 | 0.0037 | | | |
| | | | P24268 | 0.27 | 0.0001 | | | |
| | | | P80204 | 0.27 | 0.0280 | | | |
| | | | Q66HG4 | 0.27 | 0.0002 | | | |
| | | | P70490 | 0.26 | 0.0000 | | | |
| | | | P35467 | 0.26 | 0.0002 | | | |
| | | | Q9Z1Y3 | 0.26 | 0.0000 | | | |
| | | | P19939 | 0.26 | 0.0023 | | | |
| | | | P04906 | 0.26 | 0.0000 | | | |
| | | | P00758 | 0.26 | 0.0007 | | | |
| | | | Q00238 | 0.26 | 0.0019 | | | |
| | | | Q5FVR0 | 0.26 | 0.0216 | | | |
| | | | Q66H12 | 0.26 | 0.0005 | | | |
| | | | P14630 | 0.25 | 0.0047 | | | |
| | | | Q8CHN3 | 0.25 | 0.0014 | | | |
| | | | Q63083 | 0.25 | 0.0001 | | | |
| | | | Q499T2 | 0.25 | 0.0000 | | | |
| | | | Q6AYT0 | 0.25 | 0.0011 | | | |
| | | | P30919 | 0.25 | 0.0003 | | | |
| | | | O55006 | 0.24 | 0.0002 | | | |
| | | | P83121 | 0.24 | 0.0001 | | | |
| | | | P10758 | 0.24 | 0.0018 | | | |
| | | | Q4QQW8 | 0.24 | 0.0003 | | | |
| | | | Q6IMF3 | 0.24 | 0.0013 | | | |
| | | | Q9R0J8 | 0.24 | 0.0054 | | | |
| | | | P04762 | 0.23 | 0.0001 | | | |
| | | | P42854 | 0.23 | 0.0162 | | | |
| | | | Q6P6Q2 | 0.23 | 0.0001 | | | |
| | | | Q91XN4 | 0.23 | 0.0001 | | | |
| | | | Q5FVR3 | 0.23 | 0.0058 | | | |
| | | | Q80WD0 | 0.23 | 0.0061 | | | |
| | | | P25093 | 0.23 | 0.0013 | | | |
| | | | O88917 | 0.23 | 0.0001 | | | |
| | | | Q5HZW5 | 0.22 | 0.0011 | | | |
| | | | P80067 | 0.22 | 0.0013 | | | |
| | | | Q9WUK5 | 0.22 | 0.0002 | | | |
| | | | Q1WIM3 | 0.22 | 0.0003 | | | |
| | | | Q5XI43 | 0.21 | 0.0007 | | | |
| | | | Q498S8 | 0.21 | 0.0101 | | | |

| | | | | | | | | |
|---|---|---|---|---|---|---|---|---|
| | | | Q68FR6 | 0.21 | 0.0032 | | | |
| | | | Q05695 | 0.21 | 0.0002 | | | |
| | | | Q4FZV0 | 0.21 | 0.0001 | | | |
| | | | P52796 | 0.21 | 0.0002 | | | |
| | | | P27274 | 0.21 | 0.0000 | | | |
| | | | P13852 | 0.21 | 0.0000 | | | |
| | | | Q68FP1 | 0.21 | 0.0001 | | | |
| | | | Q920A6 | 0.21 | 0.0001 | | | |
| | | | Q62632 | 0.20 | 0.0005 | | | |
| | | | P00786 | 0.20 | 0.0001 | | | |
| | | | Q9JHY1 | 0.19 | 0.0000 | | | |
| | | | Q99068 | 0.19 | 0.0011 | | | |
| | | | Q63772 | 0.19 | 0.0066 | | | |
| | | | Q9R066 | 0.19 | 0.0002 | | | |
| | | | Q9ES87 | 0.19 | 0.0001 | | | |
| | | | Q08464 | 0.19 | 0.0000 | | | |
| | | | P80202 | 0.19 | 0.0001 | | | |
| | | | P50430 | 0.19 | 0.0003 | | | |
| | | | Q80WY6 | 0.18 | 0.0000 | | | |
| | | | Q4V885 | 0.18 | 0.0009 | | | |
| | | | P04182 | 0.18 | 0.0081 | | | |
| | | | P08592 | 0.18 | 0.0002 | | | |
| | | | Q5XIE8 | 0.17 | 0.0025 | | | |
| | | | P25031 | 0.17 | 0.0103 | | | |
| | | | Q5EGZ1 | 0.17 | 0.0038 | | | |
| | | | Q6IFU7 | 0.16 | 0.0003 | | | |
| | | | Q9EQV6 | 0.16 | 0.0001 | | | |
| | | | P70709 | 0.15 | 0.0046 | | | |
| | | | Q6P7A9 | 0.15 | 0.0000 | | | |
| | | | Q9QZQ5 | 0.15 | 0.0007 | | | |
| | | | Q6P767 | 0.15 | 0.0215 | | | |
| | | | Q8K1G0 | 0.14 | 0.0002 | | | |
| | | | P02625 | 0.14 | 0.0000 | | | |
| | | | B5DFC9 | 0.14 | 0.0005 | | | |
| | | | P45479 | 0.14 | 0.0174 | | | |
| | | | P97580 | 0.13 | 0.0018 | | | |
| | | | P20611 | 0.13 | 0.0000 | | | |
| | | | Q05820 | 0.12 | 0.0048 | | | |
| | | | P29598 | 0.12 | 0.0002 | | | |
| | | | P00689 | 0.09 | 0.0000 | | | |
| | | | Q8VI04 | 0.03 | 0.0027 | | | |

C.  Differential proteins identified at different time points in rat 3.

| M4-M1 | | | M8-M4 | | | M14-M12 | | |
|---|---|---|---|---|---|---|---|---|
| Accession | Fold Change | P value | Accession | Fold Change | P value | Accession | Fold Change | P value |
| | | | | | | | | |
| P20761 | 8.39 | 0.0003 | Q99PS8 | 81.91 | 0.0001 | P08426 | 4.31 | 0.0493 |
| A9UMV8 | 6.51 | 0.0009 | Q64268 | 77.32 | 0.0000 | P08723 | 3.97 | 0.0000 |
| P19939 | 6.24 | 0.0004 | P16296 | 76.61 | 0.0049 | Q8CIZ5 | 3.74 | 0.0276 |
| P20760 | 5.96 | 0.0078 | Q811M5 | 43.31 | 0.0127 | P18292 | 3.72 | 0.0006 |
| P15999 | 5.39 | 0.0002 | P26644 | 40.75 | 0.0000 | P07861 | 3.23 | 0.0034 |
| Q30KJ2 | 5.26 | 0.0010 | P04276 | 38.95 | 0.0001 | P00697 | 3.17 | 0.0001 |
| P15399 | 5.07 | 0.0007 | D3ZTE0 | 36.71 | 0.0004 | P55091 | 3.06 | 0.0487 |
| P36374 | 4.71 | 0.0000 | Q9WUW3 | 36.31 | 0.0001 | P80020 | 2.90 | 0.0394 |
| P60571 | 4.21 | 0.0065 | P20759 | 31.99 | 0.0011 | P22282 | 2.83 | 0.0002 |
| P97580 | 4.09 | 0.0002 | P09006 | 31.93 | 0.0000 | P31430 | 2.77 | 0.0022 |
| P13635 | 4.06 | 0.0001 | Q62894 | 25.50 | 0.0000 | Q06496 | 2.60 | 0.0256 |
| P12346 | 3.78 | 0.0001 | P55314 | 24.44 | 0.0000 | P02781 | 2.59 | 0.0002 |
| Q6IE52 | 3.62 | 0.0001 | Q63416 | 24.35 | 0.0000 | P36374 | 2.30 | 0.0007 |
| P14046 | 3.60 | 0.0013 | P01026 | 24.23 | 0.0002 | P97675 | 2.26 | 0.0325 |
| P07338 | 3.50 | 0.0015 | P20761 | 23.62 | 0.0000 | P53813 | 2.25 | 0.0067 |
| P02770 | 3.16 | 0.0017 | P25236 | 23.51 | 0.0001 | P07647 | 2.17 | 0.0000 |
| P27590 | 3.15 | 0.0000 | Q99041 | 22.28 | 0.0000 | P30152 | 2.15 | 0.0010 |
| P22006 | 3.12 | 0.0020 | Q30KJ2 | 19.40 | 0.0011 | P02780 | 2.03 | 0.0047 |
| P01805 | 3.04 | 0.0428 | Q62975 | 18.88 | 0.0007 | Q91XT9 | 0.49 | 0.0161 |
| P23764 | 2.91 | 0.0010 | P08934 | 18.64 | 0.0001 | Q642A7 | 0.48 | 0.0202 |
| P19218 | 2.87 | 0.0000 | Q8CG45 | 17.61 | 0.0413 | Q5M876 | 0.47 | 0.0428 |
| Q9WUW3 | 2.84 | 0.0098 | P02651 | 17.48 | 0.0000 | Q920A6 | 0.46 | 0.0105 |
| Q03626 | 2.82 | 0.0004 | P12346 | 15.34 | 0.0000 | P02651 | 0.45 | 0.0013 |
| P23928 | 2.79 | 0.0001 | P20762 | 14.17 | 0.0000 | Q4FZU2 | 0.42 | 0.0003 |
| P18424 | 2.69 | 0.0287 | P97580 | 14.07 | 0.0000 | P85971 | 0.41 | 0.0017 |
| O88767 | 2.68 | 0.0176 | P31394 | 13.99 | 0.0018 | P07943 | 0.41 | 0.0085 |
| P14141 | 2.63 | 0.0006 | Q6L711 | 12.19 | 0.0001 | Q6IFU8 | 0.39 | 0.0015 |
| P12928 | 2.61 | 0.0000 | P13635 | 11.36 | 0.0000 | P30713 | 0.39 | 0.0206 |
| P04762 | 2.60 | 0.0005 | Q4QQV8 | 11.06 | 0.0044 | Q6IMF3 | 0.37 | 0.0002 |
| P04639 | 2.56 | 0.0006 | P02783 | 10.69 | 0.0002 | P17046 | 0.35 | 0.0269 |
| Q00715 | 2.55 | 0.0264 | P55159 | 10.43 | 0.0014 | D3Z9M3 | 0.32 | 0.0030 |
| Q9QX79 | 2.51 | 0.0003 | P04916 | 10.28 | 0.0001 | Q6P6Q2 | 0.32 | 0.0375 |
| P06399 | 2.48 | 0.0013 | Q9QX79 | 10.18 | 0.0001 | Q6IG00 | 0.31 | 0.0016 |
| P35434 | 2.46 | 0.0165 | P01805 | 10.13 | 0.0003 | Q6IFW6 | 0.31 | 0.0001 |
| P01048 | 2.44 | 0.0036 | P82450 | 10.11 | 0.0011 | Q6IG02 | 0.30 | 0.0002 |
| P61972 | 2.40 | 0.0002 | P01039 | 10.05 | 0.0011 | Q30KJ2 | 0.29 | 0.0050 |
| O35078 | 2.39 | 0.0183 | P51886 | 9.87 | 0.0028 | P15399 | 0.29 | 0.0007 |

| | | | | | | | | |
|---|---|---|---|---|---|---|---|---|
| Q63678 | 2.39 | 0.0001 | Q62930 | 9.82 | 0.0023 | Q10758 | 0.26 | 0.0139 |
| Q05175 | 2.36 | 0.0038 | P50116 | 9.81 | 0.0016 | Q6IFV4 | 0.21 | 0.0001 |
| P20759 | 2.34 | 0.0484 | P81828 | 9.01 | 0.0009 | P38438 | 0.14 | 0.0052 |
| Q6P6R2 | 2.29 | 0.0002 | Q9EQV9 | 8.81 | 0.0000 | P97580 | 0.13 | 0.0000 |
| P04182 | 2.27 | 0.0289 | Q63678 | 8.33 | 0.0094 | | | |
| P52759 | 2.26 | 0.0002 | Q07523 | 7.51 | 0.0005 | | | |
| P26772 | 2.24 | 0.0007 | P02680 | 7.41 | 0.0000 | | | |
| O89117 | 2.21 | 0.0002 | P35859 | 7.04 | 0.0003 | | | |
| Q4QQV8 | 2.20 | 0.0034 | Q63207 | 6.84 | 0.0126 | | | |
| P97532 | 2.20 | 0.0226 | P06399 | 6.79 | 0.0000 | | | |
| Q64565 | 2.18 | 0.0042 | P20788 | 6.68 | 0.0047 | | | |
| P02680 | 2.18 | 0.0001 | P24090 | 6.68 | 0.0000 | | | |
| Q6TMA8 | 2.14 | 0.0035 | P02770 | 6.53 | 0.0007 | | | |
| P55063 | 2.10 | 0.0364 | P14480 | 6.52 | 0.0004 | | | |
| P07154 | 2.08 | 0.0017 | P17475 | 6.52 | 0.0000 | | | |
| P09006 | 2.05 | 0.0001 | P20059 | 5.96 | 0.0000 | | | |
| P08932 | 2.04 | 0.0011 | P15399 | 5.61 | 0.0001 | | | |
| P62963 | 2.03 | 0.0393 | Q01177 | 5.57 | 0.0003 | | | |
| P17475 | 2.03 | 0.0027 | Q6P734 | 5.26 | 0.0001 | | | |
| Q920A6 | 2.01 | 0.0031 | P20760 | 5.24 | 0.0045 | | | |
| Q99PS8 | 0.50 | 0.0001 | P08932 | 4.80 | 0.0001 | | | |
| B5DFC9 | 0.50 | 0.0059 | P01048 | 4.60 | 0.0004 | | | |
| Q63493 | 0.49 | 0.0171 | P36953 | 4.57 | 0.0000 | | | |
| P0DP29 | 0.48 | 0.0152 | P50115 | 4.52 | 0.0004 | | | |
| Q4KLZ6 | 0.48 | 0.0020 | P53813 | 3.90 | 0.0000 | | | |
| Q80W57 | 0.48 | 0.0117 | P02767 | 3.57 | 0.0002 | | | |
| Q6AXX6 | 0.48 | 0.0126 | P01946 | 3.52 | 0.0098 | | | |
| P19132 | 0.47 | 0.0023 | P07171 | 3.44 | 0.0196 | | | |
| P22282 | 0.47 | 0.0005 | P14046 | 3.41 | 0.0001 | | | |
| P01830 | 0.47 | 0.0005 | P06866 | 3.26 | 0.0009 | | | |
| P97584 | 0.47 | 0.0290 | P55091 | 3.23 | 0.0012 | | | |
| P50137 | 0.47 | 0.0031 | Q02401 | 3.17 | 0.0229 | | | |
| P70490 | 0.47 | 0.0002 | P08649 | 3.12 | 0.0002 | | | |
| P01681 | 0.47 | 0.0431 | P01015 | 2.85 | 0.0011 | | | |
| P51635 | 0.46 | 0.0039 | P19132 | 2.85 | 0.0065 | | | |
| Q6P6S9 | 0.45 | 0.0011 | Q63313 | 2.84 | 0.0028 | | | |
| P34080 | 0.45 | 0.0170 | P17559 | 2.80 | 0.0128 | | | |
| Q8R5M3 | 0.44 | 0.0013 | P25031 | 2.63 | 0.0413 | | | |
| Q9R0T4 | 0.43 | 0.0002 | Q8CJD3 | 2.60 | 0.0475 | | | |
| P08010 | 0.42 | 0.0040 | P05545 | 2.45 | 0.0004 | | | |
| P08649 | 0.41 | 0.0016 | P11517 | 2.44 | 0.0115 | | | |
| Q66HD0 | 0.41 | 0.0362 | Q63621 | 2.43 | 0.0008 | | | |
| P06760 | 0.39 | 0.0009 | P10959 | 2.32 | 0.0035 | | | |

| | | | | | | | | |
|---|---|---|---|---|---|---|---|---|
| Q9QZQ5 | 0.39 | 0.0010 | P48199 | 2.28 | 0.0143 | | | |
| Q63751 | 0.38 | 0.0000 | P08721 | 2.25 | 0.0192 | | | |
| P50115 | 0.36 | 0.0150 | Q63135 | 2.22 | 0.0018 | | | |
| P11598 | 0.35 | 0.0012 | P08025 | 2.12 | 0.0027 | | | |
| Q68FR6 | 0.35 | 0.0180 | Q6IFV1 | 2.07 | 0.0213 | | | |
| P05197 | 0.35 | 0.0096 | P02091 | 2.06 | 0.0339 | | | |
| P35053 | 0.34 | 0.0031 | P05544 | 2.05 | 0.0237 | | | |
| P01041 | 0.34 | 0.0482 | Q4G075 | 2.02 | 0.0234 | | | |
| P24090 | 0.31 | 0.0000 | Q3KR97 | 2.00 | 0.0395 | | | |
| P07171 | 0.30 | 0.0004 | P18427 | 0.50 | 0.0200 | | | |
| P06911 | 0.28 | 0.0004 | P46413 | 0.50 | 0.0003 | | | |
| Q68FR8 | 0.26 | 0.0000 | Q63279 | 0.49 | 0.0000 | | | |
| Q99041 | 0.25 | 0.0007 | Q62740 | 0.49 | 0.0033 | | | |
| P61206 | 0.23 | 0.0000 | Q9ESG3 | 0.49 | 0.0289 | | | |
| P08937 | 0.23 | 0.0000 | P70709 | 0.49 | 0.0367 | | | |
| P25031 | 0.22 | 0.0033 | P04797 | 0.49 | 0.0285 | | | |
| P30120 | 0.21 | 0.0006 | P26051 | 0.49 | 0.0008 | | | |
| Q6IE49 | 0.21 | 0.0396 | O70513 | 0.49 | 0.0001 | | | |
| P12020 | 0.20 | 0.0002 | Q9JJ19 | 0.48 | 0.0121 | | | |
| P11517 | 0.18 | 0.0035 | P10252 | 0.48 | 0.0028 | | | |
| P35444 | 0.17 | 0.0001 | Q9Z2Y9 | 0.48 | 0.0141 | | | |
| P50116 | 0.17 | 0.0025 | Q68FQ2 | 0.48 | 0.0042 | | | |
| P30710 | 0.17 | 0.0000 | Q80W57 | 0.48 | 0.0168 | | | |
| P07897 | 0.17 | 0.0003 | O54715 | 0.47 | 0.0047 | | | |
| P17559 | 0.16 | 0.0001 | Q05820 | 0.46 | 0.0192 | | | |
| Q5GRG2 | 0.15 | 0.0000 | P47853 | 0.46 | 0.0236 | | | |
| P81828 | 0.15 | 0.0430 | P04218 | 0.46 | 0.0168 | | | |
| P47727 | 0.14 | 0.0000 | P07150 | 0.46 | 0.0079 | | | |
| P08689 | 0.12 | 0.0004 | P46720 | 0.45 | 0.0049 | | | |
| P02091 | 0.12 | 0.0000 | O88989 | 0.45 | 0.0080 | | | |
| P55054 | 0.11 | 0.0007 | O70244 | 0.44 | 0.0008 | | | |
| P01946 | 0.10 | 0.0000 | Q9Z1Y3 | 0.43 | 0.0008 | | | |
| P42854 | 0.10 | 0.0000 | P81827 | 0.43 | 0.0015 | | | |
| | | | Q9ESV6 | 0.43 | 0.0146 | | | |
| | | | Q9WUC4 | 0.43 | 0.0026 | | | |
| | | | B0BND0 | 0.43 | 0.0106 | | | |
| | | | Q9JLJ3 | 0.43 | 0.0487 | | | |
| | | | P13221 | 0.43 | 0.0286 | | | |
| | | | P85971 | 0.43 | 0.0017 | | | |
| | | | G3V7W1 | 0.43 | 0.0071 | | | |
| | | | P00731 | 0.42 | 0.0406 | | | |
| | | | P19218 | 0.42 | 0.0041 | | | |
| | | | P04897 | 0.42 | 0.0051 | | | |

| | | | | | | | | |
|---|---|---|---|---|---|---|---|---|
| | | | P00884 | 0.42 | 0.0008 | | | |
| | | | Q6DGG1 | 0.42 | 0.0006 | | | |
| | | | Q8CFN2 | 0.41 | 0.0198 | | | |
| | | | P84039 | 0.41 | 0.0170 | | | |
| | | | P27590 | 0.41 | 0.0000 | | | |
| | | | P08937 | 0.41 | 0.0003 | | | |
| | | | Q99068 | 0.41 | 0.0106 | | | |
| | | | Q499T2 | 0.41 | 0.0000 | | | |
| | | | Q63474 | 0.41 | 0.0016 | | | |
| | | | Q6P6S9 | 0.40 | 0.0127 | | | |
| | | | Q6AXS4 | 0.40 | 0.0044 | | | |
| | | | Q9R063 | 0.40 | 0.0294 | | | |
| | | | P05065 | 0.40 | 0.0065 | | | |
| | | | P55063 | 0.40 | 0.0321 | | | |
| | | | P14841 | 0.39 | 0.0000 | | | |
| | | | P23377 | 0.39 | 0.0376 | | | |
| | | | P06685 | 0.39 | 0.0132 | | | |
| | | | Q9R0T4 | 0.39 | 0.0077 | | | |
| | | | P61972 | 0.39 | 0.0002 | | | |
| | | | Q6AYD4 | 0.39 | 0.0052 | | | |
| | | | Q5HZW5 | 0.39 | 0.0027 | | | |
| | | | Q80WD0 | 0.39 | 0.0094 | | | |
| | | | O70417 | 0.39 | 0.0035 | | | |
| | | | O55006 | 0.38 | 0.0001 | | | |
| | | | Q68FT5 | 0.38 | 0.0022 | | | |
| | | | Q9QWJ9 | 0.38 | 0.0483 | | | |
| | | | P01681 | 0.38 | 0.0099 | | | |
| | | | P47820 | 0.38 | 0.0076 | | | |
| | | | Q6RY07 | 0.38 | 0.0067 | | | |
| | | | P24268 | 0.38 | 0.0036 | | | |
| | | | Q9Z0W7 | 0.38 | 0.0071 | | | |
| | | | Q641X3 | 0.38 | 0.0256 | | | |
| | | | Q91XN4 | 0.37 | 0.0041 | | | |
| | | | P80020 | 0.37 | 0.0006 | | | |
| | | | Q8VIF7 | 0.37 | 0.0317 | | | |
| | | | P98158 | 0.37 | 0.0001 | | | |
| | | | P10715 | 0.37 | 0.0343 | | | |
| | | | P50399 | 0.37 | 0.0061 | | | |
| | | | P00758 | 0.37 | 0.0005 | | | |
| | | | O88766 | 0.37 | 0.0000 | | | |
| | | | O35244 | 0.37 | 0.0005 | | | |
| | | | P28826 | 0.37 | 0.0002 | | | |
| | | | Q811X6 | 0.36 | 0.0013 | | | |

| | | | | | | | | |
|---|---|---|---|---|---|---|---|---|
| | | | P62815 | 0.36 | 0.0024 | | | |
| | | | P52759 | 0.36 | 0.0001 | | | |
| | | | P54313 | 0.36 | 0.0068 | | | |
| | | | P15943 | 0.36 | 0.0148 | | | |
| | | | P97710 | 0.36 | 0.0084 | | | |
| | | | Q711G3 | 0.36 | 0.0003 | | | |
| | | | P51907 | 0.36 | 0.0146 | | | |
| | | | P10111 | 0.36 | 0.0140 | | | |
| | | | Q63716 | 0.36 | 0.0020 | | | |
| | | | P24594 | 0.35 | 0.0001 | | | |
| | | | Q498S8 | 0.35 | 0.0099 | | | |
| | | | Q9EQV6 | 0.35 | 0.0004 | | | |
| | | | P19939 | 0.35 | 0.0082 | | | |
| | | | Q4V8K5 | 0.35 | 0.0042 | | | |
| | | | P09034 | 0.35 | 0.0079 | | | |
| | | | P48508 | 0.35 | 0.0012 | | | |
| | | | P05964 | 0.35 | 0.0354 | | | |
| | | | Q1WIM3 | 0.35 | 0.0010 | | | |
| | | | O54861 | 0.35 | 0.0012 | | | |
| | | | P26772 | 0.35 | 0.0004 | | | |
| | | | Q63270 | 0.35 | 0.0095 | | | |
| | | | Q641Z6 | 0.35 | 0.0157 | | | |
| | | | P04073 | 0.35 | 0.0001 | | | |
| | | | Q6IG00 | 0.34 | 0.0064 | | | |
| | | | Q62687 | 0.34 | 0.0001 | | | |
| | | | Q5U367 | 0.34 | 0.0276 | | | |
| | | | O35112 | 0.34 | 0.0016 | | | |
| | | | Q02974 | 0.34 | 0.0022 | | | |
| | | | Q63598 | 0.34 | 0.0202 | | | |
| | | | P00787 | 0.34 | 0.0003 | | | |
| | | | Q9QXQ0 | 0.34 | 0.0019 | | | |
| | | | P53369 | 0.34 | 0.0062 | | | |
| | | | P45479 | 0.34 | 0.0072 | | | |
| | | | Q5XIE8 | 0.34 | 0.0077 | | | |
| | | | Q64565 | 0.34 | 0.0092 | | | |
| | | | Q00657 | 0.34 | 0.0019 | | | |
| | | | P11980 | 0.34 | 0.0071 | | | |
| | | | Q91XT9 | 0.33 | 0.0412 | | | |
| | | | P48500 | 0.33 | 0.0001 | | | |
| | | | P07632 | 0.33 | 0.0013 | | | |
| | | | Q5GRG2 | 0.33 | 0.0101 | | | |
| | | | P38983 | 0.33 | 0.0021 | | | |
| | | | Q9WUK5 | 0.33 | 0.0005 | | | |

| | | | | | | | | |
|---|---|---|---|---|---|---|---|---|
| | | | Q80WY6 | 0.33 | 0.0026 | | | |
| | | | Q08406 | 0.33 | 0.0328 | | | |
| | | | P25093 | 0.33 | 0.0049 | | | |
| | | | Q675A5 | 0.33 | 0.0011 | | | |
| | | | Q5FVH2 | 0.33 | 0.0017 | | | |
| | | | P62630 | 0.33 | 0.0005 | | | |
| | | | P07522 | 0.33 | 0.0020 | | | |
| | | | Q66H12 | 0.33 | 0.0042 | | | |
| | | | Q5RKI7 | 0.32 | 0.0101 | | | |
| | | | Q8K1G0 | 0.32 | 0.0020 | | | |
| | | | P08460 | 0.32 | 0.0001 | | | |
| | | | Q9Z339 | 0.32 | 0.0002 | | | |
| | | | Q99J86 | 0.32 | 0.0000 | | | |
| | | | P04642 | 0.32 | 0.0000 | | | |
| | | | Q9R1T3 | 0.32 | 0.0253 | | | |
| | | | Q64194 | 0.32 | 0.0004 | | | |
| | | | Q68FP1 | 0.31 | 0.0003 | | | |
| | | | P04904 | 0.31 | 0.0015 | | | |
| | | | Q08464 | 0.31 | 0.0000 | | | |
| | | | P07154 | 0.31 | 0.0008 | | | |
| | | | Q6AYE5 | 0.31 | 0.0010 | | | |
| | | | P07340 | 0.31 | 0.0063 | | | |
| | | | P80202 | 0.31 | 0.0137 | | | |
| | | | O35952 | 0.30 | 0.0010 | | | |
| | | | P97532 | 0.30 | 0.0032 | | | |
| | | | P57097 | 0.30 | 0.0299 | | | |
| | | | P00786 | 0.30 | 0.0032 | | | |
| | | | Q08415 | 0.30 | 0.0001 | | | |
| | | | O54728 | 0.30 | 0.0002 | | | |
| | | | Q8CHN3 | 0.30 | 0.0013 | | | |
| | | | Q4KLZ6 | 0.30 | 0.0005 | | | |
| | | | P19112 | 0.29 | 0.0090 | | | |
| | | | P14562 | 0.29 | 0.0075 | | | |
| | | | P80254 | 0.29 | 0.0023 | | | |
| | | | P52796 | 0.29 | 0.0005 | | | |
| | | | Q6P6R2 | 0.29 | 0.0004 | | | |
| | | | Q6TMA8 | 0.29 | 0.0006 | | | |
| | | | Q6AXR4 | 0.29 | 0.0007 | | | |
| | | | P13596 | 0.28 | 0.0042 | | | |
| | | | Q64319 | 0.28 | 0.0027 | | | |
| | | | P30919 | 0.28 | 0.0048 | | | |
| | | | P01830 | 0.28 | 0.0002 | | | |
| | | | P14141 | 0.28 | 0.0011 | | | |

| | | | | | | | | |
|---|---|---|---|---|---|---|---|---|
| | | | P02650 | 0.28 | 0.0008 | | | |
| | | | Q0PMD2 | 0.28 | 0.0018 | | | |
| | | | Q568Z6 | 0.28 | 0.0006 | | | |
| | | | O35763 | 0.28 | 0.0063 | | | |
| | | | P68035 | 0.28 | 0.0010 | | | |
| | | | P07314 | 0.27 | 0.0031 | | | |
| | | | P36373 | 0.27 | 0.0006 | | | |
| | | | P15083 | 0.27 | 0.0004 | | | |
| | | | O35760 | 0.27 | 0.0005 | | | |
| | | | P97605 | 0.27 | 0.0092 | | | |
| | | | Q64604 | 0.27 | 0.0043 | | | |
| | | | P12928 | 0.27 | 0.0002 | | | |
| | | | Q4QQW8 | 0.26 | 0.0003 | | | |
| | | | Q6P7A9 | 0.26 | 0.0001 | | | |
| | | | Q920A6 | 0.26 | 0.0007 | | | |
| | | | Q05695 | 0.26 | 0.0182 | | | |
| | | | P15999 | 0.26 | 0.0024 | | | |
| | | | P80204 | 0.26 | 0.0154 | | | |
| | | | P21704 | 0.26 | 0.0000 | | | |
| | | | Q6AYS7 | 0.26 | 0.0052 | | | |
| | | | P97603 | 0.26 | 0.0006 | | | |
| | | | Q64230 | 0.26 | 0.0001 | | | |
| | | | P70490 | 0.26 | 0.0008 | | | |
| | | | P00714 | 0.26 | 0.0030 | | | |
| | | | P14740 | 0.26 | 0.0001 | | | |
| | | | Q03336 | 0.25 | 0.0000 | | | |
| | | | O70534 | 0.25 | 0.0232 | | | |
| | | | Q9EPB1 | 0.25 | 0.0006 | | | |
| | | | D3ZHA0 | 0.25 | 0.0083 | | | |
| | | | P04636 | 0.24 | 0.0019 | | | |
| | | | B2RYW9 | 0.24 | 0.0022 | | | |
| | | | Q9QZA2 | 0.24 | 0.0137 | | | |
| | | | Q68FS4 | 0.24 | 0.0018 | | | |
| | | | Q64119 | 0.24 | 0.0005 | | | |
| | | | Q6MG71 | 0.24 | 0.0115 | | | |
| | | | P04906 | 0.23 | 0.0003 | | | |
| | | | P08289 | 0.23 | 0.0010 | | | |
| | | | P51635 | 0.23 | 0.0011 | | | |
| | | | P50430 | 0.23 | 0.0001 | | | |
| | | | P19804 | 0.23 | 0.0000 | | | |
| | | | Q9R0D6 | 0.23 | 0.0002 | | | |
| | | | P08010 | 0.23 | 0.0006 | | | |

| | | | | | | | | |
|---|---|---|---|---|---|---|---|---|
| | | | P11348 | 0.23 | 0.0006 | | | |
| | | | P18757 | 0.23 | 0.0005 | | | |
| | | | Q63041 | 0.23 | 0.0001 | | | |
| | | | P15684 | 0.22 | 0.0001 | | | |
| | | | P15978 | 0.22 | 0.0001 | | | |
| | | | P27274 | 0.22 | 0.0000 | | | |
| | | | P43303 | 0.22 | 0.0000 | | | |
| | | | P68370 | 0.22 | 0.0005 | | | |
| | | | P40241 | 0.22 | 0.0023 | | | |
| | | | Q63751 | 0.22 | 0.0002 | | | |
| | | | P63018 | 0.22 | 0.0022 | | | |
| | | | Q9JLS4 | 0.21 | 0.0035 | | | |
| | | | Q63424 | 0.21 | 0.0022 | | | |
| | | | Q9JI92 | 0.21 | 0.0003 | | | |
| | | | P08592 | 0.21 | 0.0083 | | | |
| | | | P50123 | 0.21 | 0.0001 | | | |
| | | | O70489 | 0.21 | 0.0006 | | | |
| | | | P00502 | 0.21 | 0.0000 | | | |
| | | | P17164 | 0.21 | 0.0117 | | | |
| | | | P31977 | 0.21 | 0.0015 | | | |
| | | | Q5XI43 | 0.20 | 0.0012 | | | |
| | | | Q9JJ40 | 0.20 | 0.0002 | | | |
| | | | P97546 | 0.20 | 0.0017 | | | |
| | | | P07861 | 0.20 | 0.0011 | | | |
| | | | P17046 | 0.20 | 0.0057 | | | |
| | | | Q64573 | 0.19 | 0.0000 | | | |
| | | | P19468 | 0.19 | 0.0005 | | | |
| | | | P0DP29 | 0.19 | 0.0273 | | | |
| | | | Q99MA2 | 0.19 | 0.0000 | | | |
| | | | P10758 | 0.19 | 0.0009 | | | |
| | | | Q64602 | 0.19 | 0.0000 | | | |
| | | | Q00238 | 0.18 | 0.0020 | | | |
| | | | P04764 | 0.18 | 0.0003 | | | |
| | | | P11232 | 0.18 | 0.0003 | | | |
| | | | P19223 | 0.17 | 0.0000 | | | |
| | | | P38918 | 0.17 | 0.0001 | | | |
| | | | P46953 | 0.17 | 0.0000 | | | |
| | | | Q5U2Q3 | 0.17 | 0.0001 | | | |
| | | | P02761 | 0.17 | 0.0000 | | | |
| | | | Q812E9 | 0.17 | 0.0010 | | | |
| | | | Q63083 | 0.16 | 0.0001 | | | |
| | | | Q9R066 | 0.16 | 0.0007 | | | |
| | | | Q66HG4 | 0.16 | 0.0001 | | | |

| | | | | | | | | |
|---|---|---|---|---|---|---|---|---|
| | | | P04762 | 0.16 | 0.0000 | | | |
| | | | Q62632 | 0.16 | 0.0012 | | | |
| | | | Q6P767 | 0.16 | 0.0007 | | | |
| | | | P62898 | 0.16 | 0.0001 | | | |
| | | | Q6P7S1 | 0.15 | 0.0000 | | | |
| | | | P41562 | 0.15 | 0.0004 | | | |
| | | | Q06496 | 0.15 | 0.0021 | | | |
| | | | P29598 | 0.15 | 0.0000 | | | |
| | | | Q4V885 | 0.15 | 0.0003 | | | |
| | | | P04182 | 0.15 | 0.0005 | | | |
| | | | Q9JHY1 | 0.14 | 0.0005 | | | |
| | | | O88767 | 0.14 | 0.0049 | | | |
| | | | Q62867 | 0.14 | 0.0009 | | | |
| | | | Q3KRC4 | 0.13 | 0.0008 | | | |
| | | | Q6AYT0 | 0.13 | 0.0026 | | | |
| | | | P68255 | 0.13 | 0.0002 | | | |
| | | | P22057 | 0.12 | 0.0000 | | | |
| | | | Q04807 | 0.12 | 0.0003 | | | |
| | | | P20611 | 0.12 | 0.0000 | | | |
| | | | P08723 | 0.12 | 0.0002 | | | |
| | | | P02625 | 0.12 | 0.0000 | | | |
| | | | Q9R0J8 | 0.12 | 0.0000 | | | |
| | | | Q6RUV5 | 0.12 | 0.0000 | | | |
| | | | P63102 | 0.11 | 0.0001 | | | |
| | | | Q4FZV0 | 0.11 | 0.0001 | | | |
| | | | B5DFC9 | 0.11 | 0.0006 | | | |
| | | | P22283 | 0.10 | 0.0015 | | | |
| | | | Q9JIK1 | 0.10 | 0.0026 | | | |
| | | | Q6P9T8 | 0.10 | 0.0003 | | | |
| | | | P02780 | 0.09 | 0.0008 | | | |
| | | | P0CG51;P62982;P62986;Q63429 | 0.09 | 0.0013 | | | |
| | | | P07647 | 0.08 | 0.0000 | | | |
| | | | Q9WTW7 | 0.08 | 0.0006 | | | |
| | | | P31044 | 0.08 | 0.0000 | | | |
| | | | P36374 | 0.07 | 0.0000 | | | |
| | | | P22282 | 0.06 | 0.0000 | | | |
| | | | Q9WTQ2 | 0.06 | 0.0001 | | | |
| | | | P02782 | 0.06 | 0.0000 | | | |
| | | | P12020 | 0.06 | 0.0000 | | | |
| | | | P02781 | 0.06 | 0.0000 | | | |

| | | | | | | | | |
|---|---|---|---|---|---|---|---|---|
| | | | P00689 | 0.06 | 0.0000 | | | |
| | | | P06911 | 0.05 | 0.0000 | | | |
| | | | Q63772 | 0.03 | 0.0051 | | | |

## D. Differential proteins identified at different time points in rat 4.

| **M4-M1** | | | **M8-M4** | | | **M14-M12** | | |
|---|---|---|---|---|---|---|---|---|
| **Accession** | **Fold Change** | **P value** | **Accession** | **Fold Change** | **P value** | **Accession** | **Fold Change** | **P value** |
| P36374 | 11.98 | 0.0000 | Q6IE49 | 111.10 | 0.0000 | P08937 | 8.37 | 0.0005 |
| Q78P75 | 10.22 | 0.0077 | P97580 | 47.51 | 0.0000 | P97574 | 5.95 | 0.0103 |
| Q68FR8 | 7.70 | 0.0001 | P15399 | 41.41 | 0.0051 | Q63751 | 5.36 | 0.0017 |
| P06760 | 7.67 | 0.0000 | Q30KJ2 | 29.05 | 0.0024 | P01946 | 5.07 | 0.0000 |
| P22006 | 6.80 | 0.0003 | P55159 | 14.52 | 0.0005 | P30152 | 4.99 | 0.0085 |
| O88767 | 6.59 | 0.0000 | P13635 | 12.75 | 0.0001 | Q9QZK9 | 4.61 | 0.0037 |
| P20761 | 6.58 | 0.0000 | P12346 | 12.70 | 0.0000 | P02091 | 4.51 | 0.0000 |
| P19629 | 6.25 | 0.0011 | P01026 | 10.68 | 0.0002 | P11517 | 4.32 | 0.0005 |
| P31044 | 5.95 | 0.0010 | Q62975 | 10.32 | 0.0000 | Q6RY07 | 3.88 | 0.0027 |
| P10715 | 5.95 | 0.0014 | P04276 | 9.44 | 0.0000 | B0BNN3 | 3.78 | 0.0024 |
| P0DMW0 | 5.44 | 0.0000 | P09006 | 9.33 | 0.0000 | Q9QZQ5 | 3.74 | 0.0000 |
| P00714 | 5.32 | 0.0011 | P55314 | 9.24 | 0.0001 | P30710 | 3.69 | 0.0061 |
| P30710 | 5.21 | 0.0000 | P14272 | 8.61 | 0.0267 | Q6IUU3 | 3.51 | 0.0000 |
| Q6P9T8 | 4.82 | 0.0028 | P08934 | 8.03 | 0.0001 | Q99041 | 3.40 | 0.0030 |
| P05964 | 4.37 | 0.0000 | P17475 | 7.37 | 0.0001 | Q924B5 | 3.28 | 0.0008 |
| Q68FR6 | 4.31 | 0.0234 | P01048 | 7.28 | 0.0000 | P97840 | 3.24 | 0.0005 |
| P20760 | 4.27 | 0.0004 | P02783 | 7.21 | 0.0007 | P27274 | 3.21 | 0.0004 |
| P68370 | 4.25 | 0.0001 | P20760 | 7.12 | 0.0000 | Q4FZV0 | 3.11 | 0.0024 |
| P07338 | 4.00 | 0.0013 | Q99041 | 6.96 | 0.0001 | P17046 | 2.91 | 0.0016 |
| Q6AXX6 | 3.89 | 0.0028 | P22006 | 6.73 | 0.0007 | Q06496 | 2.87 | 0.0061 |
| P27590 | 3.73 | 0.0001 | P01805 | 6.58 | 0.0000 | P06911 | 2.86 | 0.0074 |
| Q9R063 | 3.61 | 0.0030 | P02770 | 6.43 | 0.0060 | O88766 | 2.64 | 0.0004 |
| P06911 | 3.48 | 0.0001 | Q9QX79 | 6.34 | 0.0000 | Q5FVR0 | 2.64 | 0.0021 |
| Q924B5 | 3.25 | 0.0004 | P08932 | 6.33 | 0.0000 | P12020 | 2.59 | 0.0103 |
| P63322 | 3.25 | 0.0133 | Q4QQV8 | 6.32 | 0.0015 | P09656 | 2.58 | 0.0009 |
| Q6IFV4 | 3.19 | 0.0010 | P08721 | 6.23 | 0.0001 | P35577 | 2.58 | 0.0036 |
| P27867 | 3.16 | 0.0005 | Q01177 | 5.99 | 0.0000 | P22282 | 2.58 | 0.0006 |
| P28073 | 3.12 | 0.0015 | P21744 | 5.93 | 0.0041 | P36375 | 2.53 | 0.0087 |
| P00774 | 3.05 | 0.0243 | Q99PS8 | 5.78 | 0.0005 | Q9JLS4 | 2.50 | 0.0151 |
| P12020 | 3.04 | 0.0002 | P14046 | 5.44 | 0.0000 | P15083 | 2.49 | 0.0004 |
| P08426 | 3.03 | 0.0486 | Q63416 | 5.23 | 0.0000 | O70417 | 2.48 | 0.0077 |
| P05371 | 2.92 | 0.0002 | Q62930 | 5.20 | 0.0000 | G3V686 | 2.42 | 0.0386 |
| P01946 | 2.86 | 0.0267 | Q6IFU7 | 5.18 | 0.0006 | P29598 | 2.42 | 0.0080 |
| P14141 | 2.85 | 0.0000 | P20761 | 4.80 | 0.0000 | P47967 | 2.40 | 0.0002 |

| | | | | | | | | |
|---|---|---|---|---|---|---|---|---|
| P05065 | 2.79 | 0.0025 | Q6IE52 | 4.73 | 0.0036 | Q6P767 | 2.31 | 0.0172 |
| P19218 | 2.73 | 0.0002 | P21743 | 4.69 | 0.0105 | Q68FR6 | 2.29 | 0.0219 |
| P01805 | 2.66 | 0.0054 | P20759 | 4.56 | 0.0000 | Q6AXR4 | 2.29 | 0.0078 |
| P36373 | 2.66 | 0.0003 | Q03191 | 4.11 | 0.0022 | Q62740 | 2.27 | 0.0247 |
| P08010 | 2.66 | 0.0052 | Q63678 | 4.08 | 0.0001 | P07647 | 2.21 | 0.0000 |
| Q8CJD3 | 2.63 | 0.0049 | Q63207 | 4.08 | 0.0023 | P00714 | 2.20 | 0.0060 |
| Q6RUV5 | 2.61 | 0.0000 | Q811M5 | 4.03 | 0.0105 | B5DFC9 | 2.12 | 0.0043 |
| O89117 | 2.59 | 0.0049 | P06866 | 3.92 | 0.0002 | Q63493 | 2.11 | 0.0256 |
| P07150 | 2.56 | 0.0011 | P80204 | 3.89 | 0.0051 | Q5XI43 | 2.10 | 0.0077 |
| P07943 | 2.54 | 0.0002 | P25236 | 3.83 | 0.0000 | P01048 | 2.07 | 0.0001 |
| P50115 | 2.53 | 0.0002 | P36953 | 3.80 | 0.0009 | Q6MG71 | 2.06 | 0.0048 |
| P02780 | 2.53 | 0.0071 | P18424 | 3.76 | 0.0391 | P06760 | 2.04 | 0.0029 |
| P55054 | 2.52 | 0.0038 | P02767 | 3.71 | 0.0000 | P36374 | 2.03 | 0.0012 |
| Q6IG00 | 2.50 | 0.0107 | P51886 | 3.62 | 0.0155 | Q0PMD2 | 2.03 | 0.0133 |
| P01835 | 2.50 | 0.0234 | P24090 | 3.42 | 0.0005 | P31211 | 0.50 | 0.0004 |
| P16290 | 2.47 | 0.0001 | Q62894 | 3.41 | 0.0169 | P15473 | 0.48 | 0.0069 |
| P47820 | 2.46 | 0.0001 | Q05175 | 3.39 | 0.0008 | P09034 | 0.48 | 0.0144 |
| Q66H71 | 2.44 | 0.0109 | P05544 | 3.39 | 0.0063 | P82450 | 0.47 | 0.0123 |
| P02782 | 2.34 | 0.0003 | Q9WUW3 | 3.34 | 0.0349 | Q9QX79 | 0.47 | 0.0000 |
| P20759 | 2.32 | 0.0037 | Q63621 | 3.33 | 0.0003 | Q6P6Q2 | 0.47 | 0.0086 |
| P07647 | 2.31 | 0.0000 | P05545 | 3.21 | 0.0005 | P62836 | 0.46 | 0.0108 |
| P02625 | 2.25 | 0.0008 | Q8CG45 | 3.18 | 0.0338 | P04916 | 0.46 | 0.0006 |
| P55091 | 2.20 | 0.0024 | P00774 | 3.16 | 0.0298 | P47727 | 0.45 | 0.0011 |
| P61589 | 2.20 | 0.0065 | P31211 | 3.16 | 0.0001 | P31394 | 0.45 | 0.0010 |
| P02091 | 2.15 | 0.0396 | P00714 | 3.05 | 0.0018 | Q91XT9 | 0.45 | 0.0003 |
| P23764 | 2.15 | 0.0023 | P06399 | 3.03 | 0.0000 | P47853 | 0.44 | 0.0249 |
| Q5GRG2 | 2.11 | 0.0003 | P82450 | 3.01 | 0.0022 | Q07523 | 0.44 | 0.0084 |
| P20767 | 2.08 | 0.0039 | Q9EQV9 | 2.95 | 0.0041 | P06399 | 0.43 | 0.0013 |
| A9UMV8 | 2.06 | 0.0004 | D3ZTE0 | 2.83 | 0.0007 | Q9WTW7 | 0.43 | 0.0366 |
| Q8CFN2 | 2.03 | 0.0024 | P08649 | 2.83 | 0.0025 | Q6P6V0 | 0.42 | 0.0063 |
| Q6IFW6 | 2.01 | 0.0014 | P02680 | 2.80 | 0.0000 | Q4QQV8 | 0.42 | 0.0016 |
| P08723 | 2.00 | 0.0003 | P02651 | 2.76 | 0.0104 | P51886 | 0.41 | 0.0063 |
| P51635 | 0.50 | 0.0050 | Q62740 | 2.69 | 0.0001 | P24090 | 0.41 | 0.0018 |
| Q6P7S1 | 0.50 | 0.0018 | A9UMV8 | 2.59 | 0.0004 | P09527 | 0.40 | 0.0043 |
| Q6AYP5 | 0.50 | 0.0019 | P00762 | 2.47 | 0.0091 | P07895 | 0.40 | 0.0027 |
| P00502 | 0.49 | 0.0005 | P02764 | 2.47 | 0.0000 | P53813 | 0.39 | 0.0025 |
| Q5EGZ1 | 0.49 | 0.0033 | D4A1J4 | 2.46 | 0.0332 | P16296 | 0.39 | 0.0000 |
| Q08415 | 0.49 | 0.0024 | P10959 | 2.41 | 0.0044 | P07338 | 0.38 | 0.0007 |
| P51647 | 0.49 | 0.0028 | O70513 | 2.40 | 0.0000 | Q05175 | 0.35 | 0.0059 |
| P20786 | 0.48 | 0.0029 | P04639 | 2.38 | 0.0000 | Q6IFU8 | 0.35 | 0.0006 |
| Q812E9 | 0.48 | 0.0000 | Q6P734 | 2.37 | 0.0032 | P05545 | 0.34 | 0.0004 |
| Q711G3 | 0.48 | 0.0101 | Q6P6Q2 | 2.27 | 0.0007 | Q8CJD3 | 0.34 | 0.0199 |
| Q68FQ2 | 0.48 | 0.0028 | P62804 | 2.15 | 0.0004 | P05544 | 0.31 | 0.0394 |

| | | | | | | | | |
|---|---|---|---|---|---|---|---|---|
| Q00657 | 0.48 | 0.0000 | P26644 | 2.11 | 0.0010 | Q6IFV4 | 0.30 | 0.0045 |
| P50123 | 0.47 | 0.0004 | P53813 | 2.10 | 0.0069 | Q6IMF3 | 0.29 | 0.0010 |
| P61459 | 0.47 | 0.0005 | P07483 | 2.06 | 0.0200 | P55159 | 0.28 | 0.0019 |
| P19468 | 0.47 | 0.0000 | P20059 | 2.04 | 0.0006 | Q6IFU7 | 0.27 | 0.0108 |
| O88917 | 0.46 | 0.0164 | Q9JHY1 | 0.49 | 0.0022 | P22006 | 0.25 | 0.0017 |
| P28037 | 0.45 | 0.0028 | Q6P6S9 | 0.49 | 0.0200 | P04639 | 0.25 | 0.0005 |
| P26051 | 0.45 | 0.0078 | Q66H71 | 0.49 | 0.0055 | Q6IFW6 | 0.25 | 0.0010 |
| P08721 | 0.45 | 0.0004 | Q6AYS7 | 0.49 | 0.0080 | Q6IG02 | 0.22 | 0.0001 |
| Q5XI43 | 0.44 | 0.0172 | O35760 | 0.49 | 0.0027 | Q6IG00 | 0.22 | 0.0004 |
| P02692 | 0.44 | 0.0008 | P22057 | 0.49 | 0.0002 | Q64119 | 0.22 | 0.0070 |
| Q498S8 | 0.43 | 0.0227 | Q8K1G0 | 0.48 | 0.0466 | P97580 | 0.20 | 0.0021 |
| Q641Z6 | 0.43 | 0.0000 | Q9QXQ0 | 0.48 | 0.0055 | Q63207 | 0.20 | 0.0004 |
| P00762 | 0.42 | 0.0015 | Q63493 | 0.48 | 0.0156 | P10354 | 0.19 | 0.0026 |
| P25113 | 0.42 | 0.0019 | P04785 | 0.47 | 0.0021 | P15399 | 0.16 | 0.0016 |
| Q99041 | 0.42 | 0.0013 | P06761 | 0.46 | 0.0020 | P08025 | 0.14 | 0.0043 |
| Q9ESG3 | 0.42 | 0.0010 | Q9QZA2 | 0.46 | 0.0021 | Q30KJ2 | 0.13 | 0.0002 |
| Q9WUW9 | 0.41 | 0.0082 | P04904 | 0.46 | 0.0007 | P19814 | 0.04 | 0.0031 |
| P04073 | 0.41 | 0.0012 | Q6P7A9 | 0.45 | 0.0008 | P02651 | 0.03 | 0.0032 |
| Q4KLZ6 | 0.40 | 0.0026 | Q9JLS4 | 0.45 | 0.0069 | | | |
| Q0PMD2 | 0.39 | 0.0002 | P23764 | 0.45 | 0.0034 | | | |
| Q6P6S9 | 0.39 | 0.0003 | P46953 | 0.44 | 0.0036 | | | |
| Q9QZQ5 | 0.38 | 0.0000 | P42854 | 0.44 | 0.0455 | | | |
| P97580 | 0.37 | 0.0010 | P13221 | 0.44 | 0.0019 | | | |
| P13265 | 0.37 | 0.0104 | P48037 | 0.44 | 0.0009 | | | |
| B5DFC9 | 0.37 | 0.0028 | P08592 | 0.44 | 0.0014 | | | |
| P09656 | 0.36 | 0.0470 | P30904 | 0.44 | 0.0422 | | | |
| Q62687 | 0.36 | 0.0001 | Q64565 | 0.44 | 0.0295 | | | |
| Q63424 | 0.35 | 0.0005 | Q6AXR4 | 0.44 | 0.0016 | | | |
| Q9WUW8 | 0.35 | 0.0005 | P80254 | 0.43 | 0.0359 | | | |
| Q9JJ40 | 0.34 | 0.0008 | O35217 | 0.43 | 0.0003 | | | |
| P13596 | 0.34 | 0.0158 | P85973 | 0.43 | 0.0075 | | | |
| P00731 | 0.33 | 0.0041 | Q66HG4 | 0.43 | 0.0130 | | | |
| Q8R5M3 | 0.32 | 0.0000 | P07150 | 0.43 | 0.0013 | | | |
| Q64602 | 0.32 | 0.0001 | P07861 | 0.43 | 0.0001 | | | |
| Q5RKI7 | 0.31 | 0.0002 | P63018 | 0.42 | 0.0018 | | | |
| Q64319 | 0.29 | 0.0000 | Q9R0T4 | 0.42 | 0.0025 | | | |
| P24090 | 0.29 | 0.0000 | Q562C9 | 0.42 | 0.0075 | | | |
| Q62786 | 0.28 | 0.0119 | P00502 | 0.41 | 0.0031 | | | |
| P07171 | 0.28 | 0.0007 | P62898 | 0.40 | 0.0000 | | | |
| Q9EPF2 | 0.28 | 0.0049 | P80067 | 0.40 | 0.0059 | | | |
| Q9WTW7 | 0.27 | 0.0004 | P40241 | 0.40 | 0.0013 | | | |
| P23593 | 0.26 | 0.0045 | P48500 | 0.40 | 0.0004 | | | |
| P42123 | 0.25 | 0.0004 | Q32KJ6 | 0.40 | 0.0019 | | | |

| | | | | | | | | |
|---|---|---|---|---|---|---|---|---|
| P46720 | 0.25 | 0.0062 | Q6AYT0 | 0.40 | 0.0004 | | | |
| P42854 | 0.24 | 0.0000 | B2RYW9 | 0.39 | 0.0100 | | | |
| P35444 | 0.20 | 0.0001 | Q4FZV0 | 0.39 | 0.0006 | | | |
| P17559 | 0.20 | 0.0005 | P61206 | 0.38 | 0.0376 | | | |
| P35053 | 0.19 | 0.0006 | P27867 | 0.38 | 0.0021 | | | |
| P07897 | 0.16 | 0.0001 | P04762 | 0.37 | 0.0004 | | | |
| | | | P18418 | 0.37 | 0.0023 | | | |
| | | | Q9Z2Y9 | 0.37 | 0.0272 | | | |
| | | | Q9R0J8 | 0.37 | 0.0018 | | | |
| | | | P28073 | 0.36 | 0.0031 | | | |
| | | | O70417 | 0.36 | 0.0000 | | | |
| | | | Q5U2Q3 | 0.36 | 0.0008 | | | |
| | | | P07895 | 0.36 | 0.0015 | | | |
| | | | Q811X6 | 0.36 | 0.0071 | | | |
| | | | P84039 | 0.36 | 0.0013 | | | |
| | | | P31977 | 0.36 | 0.0034 | | | |
| | | | Q05820 | 0.35 | 0.0015 | | | |
| | | | Q8VIF7 | 0.35 | 0.0176 | | | |
| | | | P62836 | 0.34 | 0.0070 | | | |
| | | | P00884 | 0.34 | 0.0001 | | | |
| | | | O88767 | 0.34 | 0.0004 | | | |
| | | | P08937 | 0.33 | 0.0002 | | | |
| | | | P62815 | 0.33 | 0.0004 | | | |
| | | | P36373 | 0.33 | 0.0003 | | | |
| | | | P11598 | 0.32 | 0.0007 | | | |
| | | | P08723 | 0.32 | 0.0001 | | | |
| | | | P17164 | 0.32 | 0.0088 | | | |
| | | | Q63751 | 0.32 | 0.0003 | | | |
| | | | P09527 | 0.31 | 0.0086 | | | |
| | | | P04764 | 0.31 | 0.0001 | | | |
| | | | P50137 | 0.31 | 0.0004 | | | |
| | | | P01681 | 0.30 | 0.0004 | | | |
| | | | D4A5U3 | 0.30 | 0.0007 | | | |
| | | | P46462 | 0.30 | 0.0041 | | | |
| | | | P68255 | 0.30 | 0.0007 | | | |
| | | | P51635 | 0.30 | 0.0014 | | | |
| | | | P04182 | 0.30 | 0.0333 | | | |
| | | | P02781 | 0.29 | 0.0008 | | | |
| | | | Q3MIE4 | 0.29 | 0.0003 | | | |
| | | | P04642 | 0.29 | 0.0008 | | | |
| | | | Q62635 | 0.29 | 0.0208 | | | |
| | | | P41562 | 0.28 | 0.0016 | | | |
| | | | P30120 | 0.28 | 0.0002 | | | |

| | | | | | | | | |
|---|---|---|---|---|---|---|---|---|
| | | | P47820 | 0.27 | 0.0000 | | | |
| | | | G3V7W1 | 0.27 | 0.0000 | | | |
| | | | P05964 | 0.27 | 0.0000 | | | |
| | | | P02782 | 0.27 | 0.0001 | | | |
| | | | P05065 | 0.26 | 0.0012 | | | |
| | | | P29598 | 0.26 | 0.0004 | | | |
| | | | Q6GMN2 | 0.26 | 0.0026 | | | |
| | | | P68370 | 0.26 | 0.0001 | | | |
| | | | P61589 | 0.26 | 0.0014 | | | |
| | | | P19804 | 0.25 | 0.0000 | | | |
| | | | P24368 | 0.25 | 0.0000 | | | |
| | | | Q6IUU3 | 0.25 | 0.0001 | | | |
| | | | Q9QZK9 | 0.24 | 0.0000 | | | |
| | | | Q9EQX9 | 0.24 | 0.0008 | | | |
| | | | Q3KR97 | 0.24 | 0.0002 | | | |
| | | | P22283 | 0.24 | 0.0002 | | | |
| | | | P0DMW0 | 0.23 | 0.0001 | | | |
| | | | P50399 | 0.23 | 0.0036 | | | |
| | | | P97605 | 0.23 | 0.0026 | | | |
| | | | P02780 | 0.23 | 0.0028 | | | |
| | | | P45479 | 0.23 | 0.0003 | | | |
| | | | P53369 | 0.23 | 0.0006 | | | |
| | | | P07647 | 0.22 | 0.0000 | | | |
| | | | Q6AXX6 | 0.21 | 0.0018 | | | |
| | | | P08025 | 0.21 | 0.0042 | | | |
| | | | P01041 | 0.21 | 0.0007 | | | |
| | | | P63102 | 0.21 | 0.0005 | | | |
| | | | P36374 | 0.21 | 0.0000 | | | |
| | | | P00689 | 0.20 | 0.0000 | | | |
| | | | Q6RUV5 | 0.19 | 0.0000 | | | |
| | | | P31044 | 0.19 | 0.0011 | | | |
| | | | P05197 | 0.19 | 0.0031 | | | |
| | | | P08010 | 0.18 | 0.0003 | | | |
| | | | Q9JI85 | 0.18 | 0.0004 | | | |
| | | | Q6P9T8 | 0.18 | 0.0024 | | | |
| | | | P22282 | 0.18 | 0.0000 | | | |
| | | | P47727 | 0.18 | 0.0009 | | | |
| | | | P55063 | 0.17 | 0.0044 | | | |
| | | | P10354 | 0.17 | 0.0012 | | | |
| | | | P10715 | 0.17 | 0.0002 | | | |
| | | | Q78P75 | 0.17 | 0.0110 | | | |
| | | | P16290 | 0.16 | 0.0001 | | | |
| | | | P30710 | 0.15 | 0.0000 | | | |

| | | | | | | | | |
|---|---|---|---|---|---|---|---|---|
| | | | Q68FR8 | 0.15 | 0.0003 | | | |
| | | | P07943 | 0.14 | 0.0000 | | | |
| | | | Q924B5 | 0.13 | 0.0000 | | | |
| | | | Q8CFN2 | 0.09 | 0.0000 | | | |
| | | | P12020 | 0.06 | 0.0001 | | | |
| | | | P06911 | 0.05 | 0.0000 | | | |
| | | | P19629 | 0.05 | 0.0006 | | | |
| | | | Q5GRG2 | 0.04 | 0.0000 | | | |
